# Physical characteristics of capacitive carbons derived from the electrolytic reduction of alkali metal carbonate molten salts†

**DOI:** 10.1039/c9ra05170h

**Published:** 2019-11-11

**Authors:** Matthew A. Hughes, Robert D. Bennett, Jessica A. Allen, Scott W. Donne

**Affiliations:** Discipline of Chemistry, University of Newcastle Callaghan NSW 2308 Australia scott.donne@newcastle.edu.au; CSIRO Energy Technology Research Way Clayton Victoria 3168 Australia; School of Engineering, Priority Research Centre for Frontier Energy Technologies and Utilisation, University of Newcastle Callaghan NSW 2308 Australia

## Abstract

Carbons have been synthesized through the reduction of molten carbonate systems under varied conditions. The mechanism and kinetics of carbon electrodeposition has been investigated. Carbon morphologies include amorphous, graphite-like, and spherical aggregate phases. Increased graphitic character is observed in carbons electrodeposited at more cathodic potentials, particularly at higher temperatures. Bonding has been investigated and oxygen functionalised sp^2^ and sp^3^ structures have been identified. The level of functionalization decreases in carbons with reduced amorphous and increased graphitic character. Thermal decomposition of electrodepositied carbons has been investigated and zero order kinetics have been identified. A relationship has been identified between elevated oxygen functionalization and increased pseudo-capacitance, with carbons deposited at 0.15 A cm^−2^ showing capacitances of 400 F g^−1^ in 0.5 M H_2_SO_4_ at sweep rates of 10 mV s^−1^.

## Abstract 2

Carbons have been synthesized through the reduction of molten carbonate systems under varied conditions.

## Graphical abstract

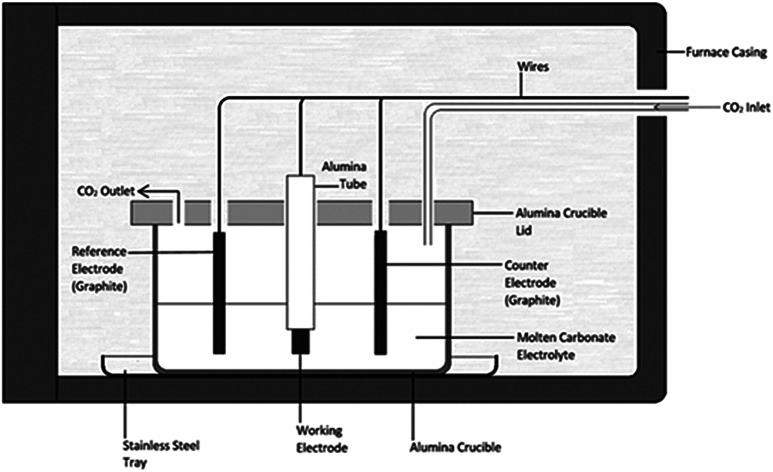

## Introduction

Carbon is vital both for existence of organic life,^1^ and for many industrial and technological applications.^1^ Carbonaceous materials are used extensively in the electrochemical production of fluorine,^2,3^ the generation of heat and electricity,^4^ the formation of gas and liquid fuels,^4^ the synthesis of drugs,^1^ the generation of plastics,^5^ solar cell construction,^6^ the manufacture of electronic devices,^7,8^ and in the production of lithium-ion batteries and supercapacitors.^7–10^ This versatility is a direct result of the diverse bonding interactions carbon exhibits.^1^ The extensive variability in the molecular and morphological properties of carbonaceous materials leads to widely varying optical properties,^6^ adsorption characteristics,^8^ electron mobilities,^8^ thermal conductivities,^8^ ion adsorption properties,^8^ and physical strengths.^8^ Due to these wide-ranging characteristics, carbons with different structures and properties are used in many industries including aerospace, automotive, biotechnology, portable and consumer electronics, and energy storage.^8^ A huge body of research has been dedicated to carbon synthesis, with both the improvement of traditional carbon synthesis methods, and the development of novel synthetic methods receiving considerable attention.

A range of methods have been used historically to prepare carbon for industrial and chemical applications, with the most common being pyrolytic,^11,12^ hydrothermal,^13,14^ chemical vapourdeposition (CVD), arc discharge, and laser ablation.^15–19^ These are capable of producing controlled carbon structures in high yields (>75%),^18^ however they are energetically expensive.^20^ As such, low cost and simple production of controlled carbon structures is a focus of intense investigation in contemporary research. Multiple alternative methods of carbon synthesis have been investigated by previous researchers, with the electrolytic reduction of molten carbonate salts standing out as a particularly interesting approach. Previous work in this area has characterized the technique as a medium temperature method of producing carbonaceous products with physical and electrochemical properties that vary considerably based on the conditions of electrodeposition.^10,21–30^ It has been demonstrated that a correlation exists between electrodeposition parameters and the structure and morphology of the resultant carbon materials.^29^ In particular, it has been found that temperature^10,26,30,31^ and substrate^32^ affect both the morphology and physical characteristics of carbons synthesized in this manner, and that the electrolyte^27,33^ and current density^34^ may also have an influence on the structure of the electrodeposited carbon.

Carbon production through the electrolytic reduction of molten carbonate salts shows multiple advantages over alternative methods. Beyond the low energy and moderate synthesis temperature requirements mentioned previously, the potential application of this method for CO_2_ sequestration and transformation has also been investigated.^31,35–38^ This application of carbonate reduction is highly desirable since CO_2_ is a major greenhouse gas and the by-product of many industrial reactions, particularly those involved in energy production from fossil fuels.^31^ A manner of converting otherwise waste CO_2_ gas into value-added carbon materials is therefore highly sought after. Beyond this, it has been shown that the carbon materials produced through molten carbonate reduction pathways act as excellent supercapacitor materials,^29,39^ with specific capacitances that vary depending upon synthesis conditions. This variation in capacitance is likely the result of variation in the morphological and physical characteristics of the materials. Therefore, this manuscript provides an in-depth investigation of variation in the structure and physical characteristics of carbon materials synthesized through the electrolytic reduction of molten carbonate salts with systematic changes in the conditions of carbon synthesis. A brief investigation of the capacitive behaviour of electrodeposited carbons and its relationship to the identified physical characteristics for the materials is also supplied.

## Experimental

### Carbon synthesis

**Fig. 1:**
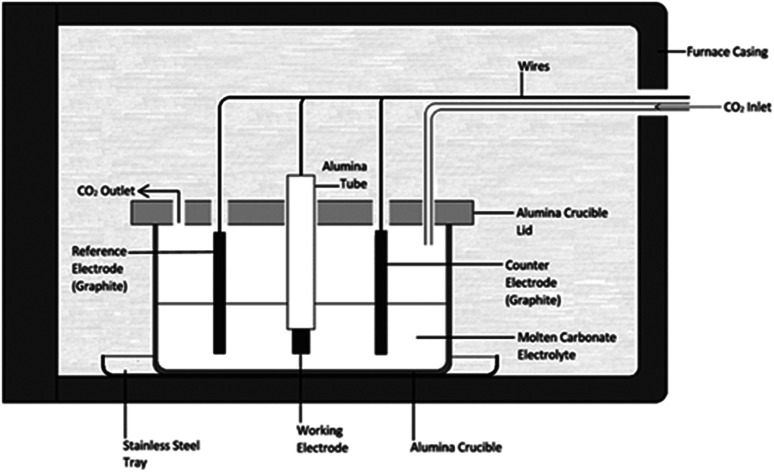
Schematic for the electrochemical cell setup used throughout this research.

Carbons were synthesized using methods previously used by this group.^28^ Three electrode cells containing molten alkali metal carbonates at eutectic compositions were used for all carbon electrodeposition experiments (Fig. 1). Molten alkali metal carbonates consisted of either Li_2_CO_3_–Na_2_CO_3_–K_2_CO_3_ (43.5 : 31.5 : 25.0 mol%, mp 397 °C),^40,41^ Li_2_CO_3_–K_2_CO_3_ (62.0 : 38.0 mol%, mp 489 °C),^41^ or Li_2_CO_3_–Na_2_CO_3_ (52.0 : 48.0 mol%, mp 500 °C).^41^ Eutectic salts were prepared from solid Li_2_CO_3_, Na_2_CO_3_ and K_2_CO_3_ precursors (Sigma-Aldrich, >99%). Prior to preparation carbonate powders were dried in air at 110 °C for at least 24 hours. Following this the powders were weighed and mixed together at the appropriate eutectic composition, initially with a mortar and pestle, then through ball-milling at 170 rpm in 10 forward and 10 reverse (2 minute) cycles using a Fritsch Pulverisette 6 stainless steel ball mill. Eutectics were fused at 500 °C for 1 hour under a 60 mL min^−1^ CO_2_ flow (BOC gases; food grade). The CO_2_ atmosphere was used to reduce carbonate decomposition and to assist in the regeneration of carbonate ions in the melt.^26^ In all cases eutectics were fused in alumina crucibles topped with an alumina lid containing holes for the placement of electrodes and a CO_2_ inlet and outlet. Alumina was chosen over other crucible materials as, in the presence of lithium-containing molten carbonates, alumina forms a corrosion layer of lithium aluminate, which increases the surface roughness of the material,^42^ but is otherwise stable. Following fusing, eutectics were allowed to cool to room temperature, prior to being re-heated for carbon electrodeposition. The mechanism and kinetics of carbonate reduction were examined using cyclic voltammetry with a WaveNow potentiostat controlled by AfterMath software prior to bulk carbon electrodeposition.

Electrodeposition experiments were carried out in the temperature range 500–700 °C (±5 °C) in a programmable LABEC ashing furnace. The heating rate used was ∼2 °C min^−1^. CO_2_ gas was allowed to flow at atmospheric pressure across the surface of the carbonate electrolyte at a rate of 60 mL min^−1^. All electrodes were held above the carbonate eutectic until it became molten, at which point they were submerged and allowed to equilibrate for 30 minutes. Working electrodes were prepared from the foils and rods (Table 1). They were prepared by sealing the substrate into an alumina tube using Resbond 989 (Ceramic Oxide Fabricators, Australia) to produce controlled surface areas which could be exposed to the carbonate melt. Counter and reference electrodes were prepared from graphitized carbon rods. The graphitic reference system, hereafter referred to as the C/CO_2_/CO_3_^2−^ system, has previously been shown to be ideal for voltammetric studies in molten carbonate media.^43^ In all cases the pre-electrodeposition geometric surface area of the working electrode exposed to the electrolyte (<2.0 cm^2^) was considerably smaller than that of the graphite counter electrode (>7.0 cm^2^), thus ensuring the cell was working electrode limited.^44^

**Table 1:** Foils and rods used in the construction of working and counter electrodes for experimental carbon deposition

Material	Form	Purity (%)	Thickness (mm)
Au	Foil	99.99	0.25
Cu	Foil	99.98	0.25
Ti	Foil	99.70	0.25
Graphite	Rod	99.99	6.75 (dia.)

Carbon electrodeposition was carried out at current densities of 0.15–1.20 A cm^−2^ relative to the geometric surface area of the working electrode. The current supplied to cells was controlled using a GW Laboratory DC power supply (GPS-1830). The potential difference between the reference electrode and the working electrode was recorded across the duration of deposition using a WaveNow potentiostat controlled by AfterMath software. Following electrodeposition, carbons were manually removed from the solidified carbonate and soaked in 1.0 M H_2_SO_4_ for at least 24 hours to remove carbonate impurities. The control conditions for carbon production, hereafter referred to as the standard conditions, were carbon electrodeposition from the ternary alkali metal carbonate eutectic onto a graphite substrate at 600 °C using a working electrode current density of 0.25 A cm^−2^. To produce carbons under a wide range of comparable conditions, singular parameters, such as electrodeposition temperature or substrate, were altered and carbon electrodeposition was allowed to proceed under these new conditions.

### Characterization

The structure of individual carbons was examined using a Phillips X'Pert MPD equipped with a copper anode (Cu K_α_*λ* = 1.5408 Å). XRD patterns were recorded in a 2*θ* range of 5–90° 2*θ* at a voltage of 40 kV and a current of 30 mA. A step size of 0.007° was used with a scan rate of 1.5° 2*θ* min^−1^. Data analysis was carried out using the X'Pert HighScore program, with peak assignment being based on information drawn from the International Centre for Diffraction Data database. Infrared spectra of carbon samples were recorded using a PerkinElmer UATR Two spectrophotometer. Spectra were collected over the interval 4000–400 cm^−1^ using 4 scans with a resolution of 2 cm^−1^. Raman spectra were recorded using an Andor HoloSpec F/1.8 spectrograph configured with a B&W Tek CleanLaze 785 nm 495 mW excitation laser operating at 50% power, and low frequency grating (HS-HSG-785-LF). Detection was *via* an iDus420CCD camera. X-ray photoelectron spectroscopy (XPS) was performed on samples which were pressed into indium foil (99.99%, thickness: 0.2 mm, CMR *direct*) using a stainless steel roller. The indium strips were fixed to sample holders using carbon tape. The filled sample holders were degassed and transferred to the XPS analysis chamber. Samples were illuminated with a non-monochromatic Al K_α_ (Omnivac, 1486.6 eV) X-ray source and the photoemission spectra was collected using a SES2002 analyser (Scienta). Survey scans were carried out using a pass energy of 100 eV, while regions scans were performed using a pass energy of 20 eV and 200 meV steps. The working pressure in the analysis chamber was typically 2.0 × 10^−9^ mBar, with a base pressure of 4 × 10^−10^ mBar. Survey scans were energetically aligned by shifting the C 1s peak to 284 eV to match CASA's internal reference for carbon. Region scans were aligned through fitting a series of peaks to the material under investigation and shifting the sp^2^ peak to 284.5 eV in accordance with the literature on graphitic carbon. SEM images of carbons were obtained using a Zeiss Sigma VP FE-SEM fitted with a Bruker light element SSD EDS detector. The carbon powders were lightly dusted onto Al sample holders covered with carbon tape. An accelerating voltage of 5 kV was used in most cases. Carbons were gold coated to reduce charging prior to imaging. Energy Dispersive X-ray Spectroscopy (EDS) analysis was carried out on points at the surface of uncoated materials using an accelerating voltage of 10 kV. The morphology of electrodeposited carbon was examined using a JEOL 2100 LaB_6_ TEM. The carbon materials were suspended in ethanol and sonicated prior to being dried on carbon-coated copper grids. The specific surface area of select carbon sample was determined from gas adsorption using a Micrometrics ASAP 2020 Surface Area and Porosity Analyzer. Carbon samples (∼0.02 g) were heated under vacuum at 300 °C for 24 h prior to analysis. Due to the high surface area and low mass of the samples this quantity of synthesized carbon was sufficient to determine the specific surface areas (SSAs) of the materials with minimal error from the sample size, with the literature indicating that, for carbons with surface areas comparable to those examined here (>100 m^2^ g^−1^), even 0.01 g can be sufficient to obtain a relatively accurate measure of BET SSA.^45^ A 9-point N_2_ adsorption isotherm was taken at 77 K over the relative pressure range 0.05–0.30 *P*/*P*_O_. This isotherm was used to determine the SSAs of the examined carbons using the linearized BET isotherm. The combustion kinetics, activation energies, and the apparent frequency factors of both electrodeposited and activated carbons were investigated through thermogravimetric analysis (TGA). Alumina crucibles loaded with ∼5 mg of carbon sample were heated from 25 °C to 850 °C using a NETZSCH STA 2500 Regulus. α-Al_2_O_3_ was used as a reference material. The samples were heated initially at 5 °C min^−1^ to 105 °C where they were held isothermally for 10 min to completely remove trace moisture from the materials. They were then heated from 105 °C to 850 °C at a heating rate of 5 °C min^−1^. Inert N_2_ gas was passed over the sample at 60 mL min^−1^ throughout the experiment.

### Electrochemical characterization

Three electrode cells were constructed using conventional methods.^28^ Such cells have been used effectively to gauge the electrochemical performance of activated carbons.^46^ After assembly cells were allowed to equilibrate at open circuit potential (OCP) for 60 minutes, following which the potential was cycled between −0.5 V to 0.3 V *vs.* SCE at a rate of 25 mV s^−1^ to establish reversible cycling (250 cycles). Analysis of CV profiles was carried out in the R2018a build of MATLAB. Following this, step potential electrochemical spectroscopy (SPECS) was performed by stepping the potential from −0.5 V to 0.3 V *vs.* SCE and back again with ±0.025 V steps followed by 300 s equilibration periods after each step. Analysis of the SPECS results was carried out in the R2018a build of MATLAB using linear least-squared error curve fitting based on the trust-region-reflective algorithm.

## Results and discussion

### Mechanistic studies

The mechanism of carbon electrodeposition from molten carbonate salts has previously been investigated,^10,30,47^ with both multi-step and single step mechanisms being proposed. Of the proposed mechanisms, the most supported is composed of a single 4-electron reduction of the carbonate ion;^48^*i.e.*,

1 CO_3_^2−^ + 4e^−^ → C + 3O^2−^

However, based on the recent identification of the pyrocarbonate ion (C_2_O_5_^2−^) as the major form taken by CO_2_ upon dissolution in molten carbonate media^38^ it is possible that, in the presence of dissolved CO_2_, a second path of carbon formation may also occur; *i.e.*,

2 C_2_O_5_^2−^ + 4e^−^ → C + 3O^2−^ + CO_2_

With rapid recombination of CO_2_ and CO_3_^2−^ to pyrocarbonate ions occurring close to the surface of the working electrode.

**Fig. 2:**
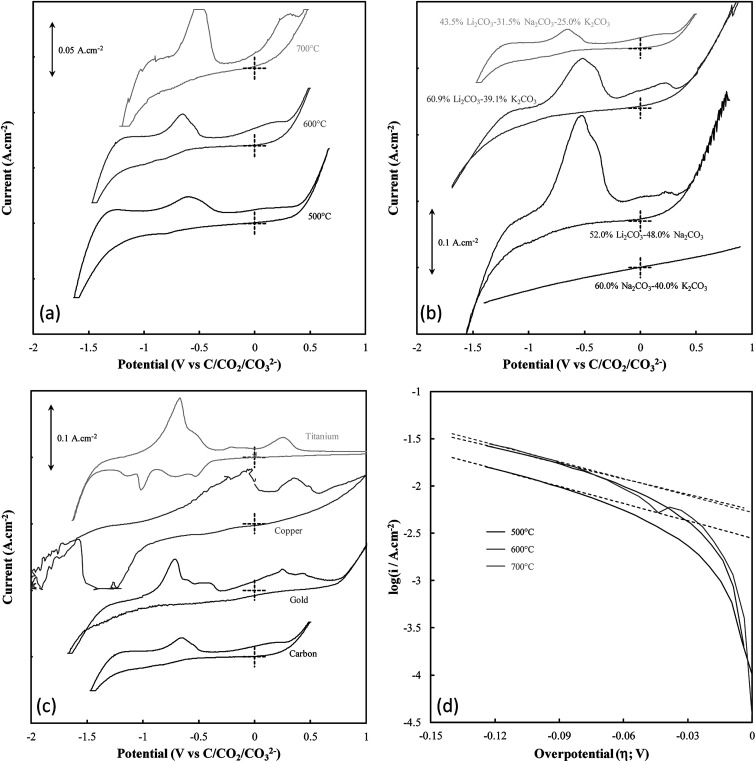
(a–c) CVs taken at 20 mV s^−1^ in molten carbonate systems with grouping based on the deposition variable under investigation, these being; (a) the temperature of carbon deposition, (b) the molten carbonate electrolyte in which deposition occurred, and (c) the substrate onto which deposition occurred. Labels are assigned to CVs based on the exact variation from standard deposition conditions. (d) Cathodic Tafel curves and the calculated Tafel lines (dotted) for the Tafel peak associated with the onset of carbon deposition for carbons deposited at temperatures in the range of 500–700 °C.

The mechanism of carbon electrodeposition from molten carbonates was investigated through CV prior to the commencement of bulk electrodeposition (Fig. 2(a)–(c)). Note that when identifying carbon electrodeposition conditions based on Fig. 2, legend entries refer to the variation from standard conditions used to synthesize a carbon, so the legend entry 600 °C, Li–K–Na, and C corresponds to the standard conditions in each of Fig. 2(a), (b), and (c), respectively.

Systems were not studied separately based on the current density of electrodeposition as, prior to the commencement of bulk electrodeposition, these systems were not in any way different from the system under standard conditions. The influence of temperature variation on the electrodeposition of carbon from ternary carbonate system has been previously addressed,^39^ with it being found that temperature influences the potential at which carbonate reduction and carbon oxidation is observed. This can be seen in Fig. 2(a) from considerable broadening of the CVs taken for carbon electrodeposition at 500 °C as compared to the CVs taken at 600 °C or 700 °C. This is potentially due to changes in the thermal energy of the system with temperature variation. Previous groups have attributed similar phenomenon to faster electron kinetics at elevated temperature.^49^ Based on this it is apparent that this variation is likely due to increased thermal energy in the system, with carbon electrodeposition potentials being less negative at elevated temperatures. This phenomenon is consistent with that expected based of the calculated reaction enthalpy of Li_2_CO_3_ reduction at 900 K, close to the temperature of reduction that was experimentally used as a standard condition (600 °C) throughout this research; *i.e.*,

3 3Li_2_CO_3_ → 3Li_2_O + 2CO_2_ + O_2_ + C

At this temperature Δ*H*^o^_r_ = 1176.3 kJ mol^−1^ (calculated from standard enthalpies of formation)^50^ which is endothermic meaning the formation of carbon will be favoured by increased temperatures.

In addition to temperature variation, it is apparent (based on Fig. 2(b) and (c), respectively) that both the electrolyte and electrodeposition substrate also influence the potential of carbon electrodeposition. The magnitude of these effects and the kinetics of carbon electrodeposition in different systems were investigated based on the cathodic Tafel curves for each system at the potential of carbon electrodeposition (shown for the case of temperature variation in Fig. 2(d)). Cathodic Tafel curves were used over their anodic counterparts as anodic slopes showed considerable divergence from ideal behaviour. This type of effect has been reported previously to be due to limitations posed to the system by mass transfer. In an ideal system, the exchange current density (*i*_0_) and charge transfer coefficients (*α*) may be extracted from these curves based on the slope and intercept of linear regions close to 0.118 V *vs.* NHE.^51^ In the examined systems this region was taken to be the maximal linear segment close to 0.1 V *vs.* C/CO_2_/CO_3_^2−^ (Table 2). Linear segments of this form are shown for carbon electrodeposition at 500 °C, 600 °C and 700 °C in Fig. 2(d). Due to subjectivity in selecting the region from which linear parameters were extracted, the trends in the kinetic parameters should be considered over the exact presented values. Based on comparison to the ideal transfer coefficient (*α* = 0.5) and exchange current density (*i*_0_ ≈ 10^−6^ A cm^−2^) for a reversible system,^51^ in all cases, the electrodeposition of carbon from molten carbonate salts shows considerable irreversible character. This contributes to the non-ideal anodic Tafel curves for the carbon electrodeposition reaction and may be due in part to the facile nature of pyrocarbonate reformation and O^2−^ recombination to oxygen imposing limitations on the availability of reactive species for the back-reactions of both eqn (1) and (2).

**Table 2:** Transfer coefficients (*α*) and exchange current densities (*i*_0_) at the onset of carbon deposition in molten carbonate systems

			*α*	*i*_0_ (mA cm^−2^)
Parameter under variation	Temp. (°C)	500	0.83	3.4
		600	0.87	6.3
		700	0.99	6.5
	Electrolyte	K–Na	NA	NA
		Li–K	0.85	4.2
		Li–Na	0.82	7.3
		Li–K–Na	0.87	6.3
	Substrate	Au	0.74	5.4
		Ti	0.92	1.1
		C	0.87	6.3
		Cu	0.47	0.7

Based on the high values of *α* presented in Table 2, the equilibrium at low overpotentials surrounding the region of carbon electrodeposition in molten carbonate salts is heavily in favour of the production of carbon, with high exchange current densities indicating that carbon is readily produced from molten carbonate media in these systems. No charge transfer coefficients or exchange current densities are reported when attempting to deposit from binary Na–K carbonate systems as carbon electrodeposition does not occur in these systems (evidenced by the featureless CV for the system presented in Fig. 2(b)). The inability for carbon electrodeposition to occur in the Na–K system is due to the absence of lithium ions in the system, which have been shown to be necessary for the electrodeposition of carbon from molten carbonate systems based on both experimental evidence^27^ and thermodynamic considerations.^10^ This is due to the relative electrodeposition potentials of metals and carbon from the molten carbonate system being such that, in the absence of lithium ions, the electrodeposition potential of metals from the molten electrolyte is lower than the potential of carbon electrodeposition.^10^ In general, based on Table 2, it is apparent that as the temperature of carbon electrodeposition increases, both *α* and *i*_0_ tend to increase. This indicates that, at elevated temperatures, electrodeposition of carbon from molten carbonates becomes increasingly favourable kinetically and thermodynamically. This is consistent with the results of our previous analysis based on the thermodynamics of carbon electrodeposition in molten carbonates. The trends present in the values of *α* and *i*_0_ with variation in electrodeposition substrate and electrolyte are less apparent than those for variation in electrodeposition temperature. This is due to the non-quantitative nature of these variables. In general, the value of *α* and *i*_0_ are largely independent of the electrolyte in which carbon electrodeposition occurs, indicating that carbon electrodeposition is equally favoured in each system. Compared to this there is considerable variation in the values of *α* and *i*_0_ with the substrate onto which carbon is electrodeposited, with an *α* value close to 0.5 indicating that carbon electrodeposition onto copper substrates may be close to reversible. This does not, however, indicate that copper is an optimal electrode for carbon electrodeposition in molten carbonate salts, with Fig. 2(c) showing that a considerable number of extra oxidation and reduction peaks exist for carbon electrodeposition onto a copper substrate as compared to a graphite substrate. Based on the relative magnitudes of *i*_0_ presented in Table 2, it is considerably less energetically intensive electrodepositing carbon onto a gold or graphitic carbon substrate than it is electrodepositing onto a titanium or copper substrate.

Beyond the features of carbon electrodeposition onset, the CVs presented in Fig. 2(a)–(c) show a diverse range of reduction and oxidation features. All CVs collected using graphitic carbon working electrodes show broadly the same features, these being the previously examined cathodic limit corresponding to carbonate reduction, the anodic limit corresponding to oxygen and carbon dioxide evolution,^31^ and two major oxidation peaks in the vicinity of −0.6 V and 0.25 V, respectively.^10,30^ Oxidation peaks close to these potentials at glassy carbon electrodes have previously been attributed to the two step oxidation of carbon formed during the reduction of molten carbonate electrolyte,^30^ with net anodic reactions of eqn (4) and (5) being proposed;^10^*i.e.*,

4 C + 2O^2−^ → CO_2_ + 4e^−^

5 C + 2CO_3_^2−^ → 3CO_2_ + 4e^−^

The CVs shown in Fig. 2(c) for the electrodeposition of carbon onto different metallic substrates shown a range of features absent during carbon electrodeposition onto graphitic carbon. Both the CVs for carbon electrodeposition onto copper and titanium substrates show multiple oxidation peaks not apparent during carbonate reduction onto graphitic substrates, and each of the CVs for carbon electrodeposition onto gold, titanium and copper substrates show anodic peaks which are absent during carbon electrodeposition onto graphite. In addition, CVs for carbon electrodeposition onto both gold and, with less certainty, copper substrates show cathodic loops (observed at ∼−1.75 V and −2.00 V, respectively). The presence of cathodic loops may be caused by growth of the working electrode over time, however, due to the low currents used to produce cyclic voltammograms, very little electrode growth is present during CV. Cathodic loops may also appear when a new phase is formed that changes the character of the working electrode surface. In this case it is likely that this phase is electrodeposited carbon. The appearance of cathodic loops may be attributed to the fact that the electrodeposition of carbon onto carbon requires a lower overpotential than the electrodeposition of carbon onto the gold or copper substrate, hence the rate of carbon electrodeposition increases slightly with both time and as the potential is swept downwards. This leads to increased oxidation currents close to the cathodic limit once the scan direction is reversed, contributing to the formation of a loop in the cyclic voltammogram for the system. A major implication of these cathodic loops is that electrode surface areas tend to increase with time as carbon is electrodeposited first onto the electrode surface, then onto the formed carbon phase itself. The absence of cathodic loops when depositing onto titanium substrates may be due to the large number of competing cathodic reactions in occurrence when using this electrode material, which leads to a significant decrease in the rate of carbon electrodeposition and so a considerable decrease in the visibility of cathodic loops.

**Fig. 3:**
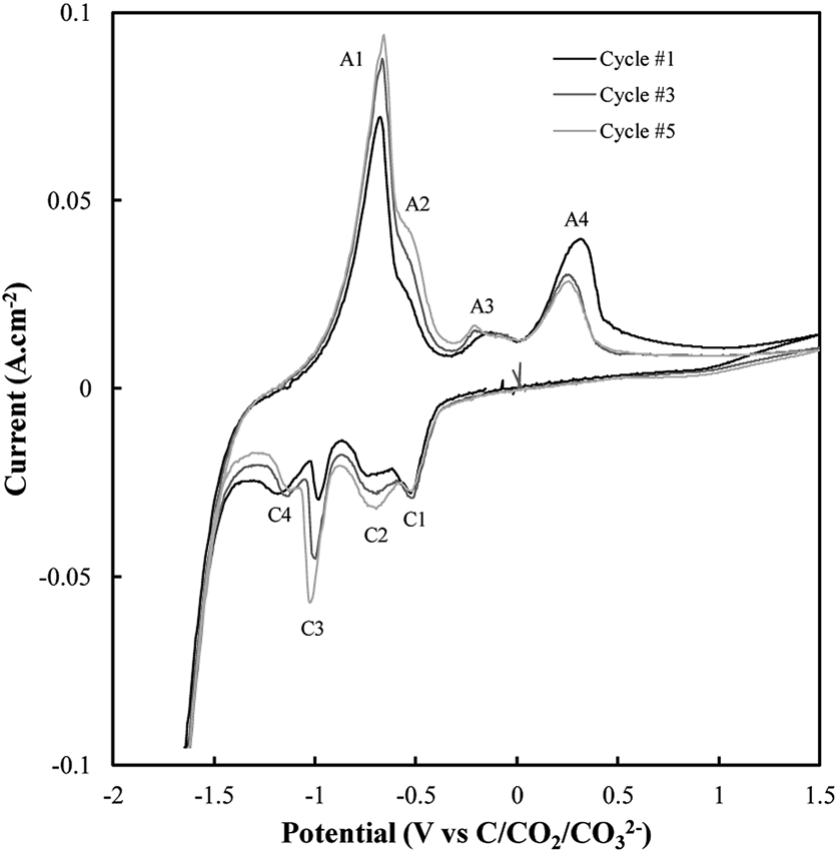
Sequential CVs taken using a sweep rate of 5 mV s^−1^ at a titanium working electrode under otherwise standard conditions (ternary carbonate, 600 °C).

Based on the CV corresponding to electrodeposition onto a titanium substrate in Fig. 2(c), there are four major reduction peaks and at least two unidentified oxidation peaks beyond what we would expect in the case of simple carbonate reduction. Since the successful electrodeposition of carbon on titanium substrates was experimentally confirmed during bulk electrodeposition experiments, several of the unidentified anodic peaks relate to the oxidation of carbon. To determine which peaks these are, and which peaks relate to undesired side-reactions, the influence of successive potential cycling on CV current densities at a titanium substrate was investigated (Fig. 3).

Based on Fig. 3, the current density close to the potential of carbon electrodeposition onset at a titanium substrate (∼−1.2 V *vs.* C/CO_2_/CO_3_^2−^) tends to decrease with increasing cycle number. This was taken to either indicate that, with increasing cycle number, side reactions dominate the reduction of carbonate to an increasing degree, or that, with increased potential cycling, the titanium substrate became less active to carbon electrodeposition. Since, during the reduction of molten carbonate salts, the magnitude of peaks related to the oxidation of electrodeposited carbons should be proportional to the amount of carbon deposited (assuming no limitations due to mass transport), the anodic peaks associated with carbon oxidation in Fig. 3 were theorized to be those with magnitudes that decreased with cycle number, specifically A3 and A4. The potentials of these peaks (∼−0.25 V and ∼0.25 V *vs.* C/CO_2_/CO_3_^2−^) are close to the previously identified potentials of carbon oxidation peaks in CVs taken at graphitic substrates. In addition to these now identified carbon oxidation peaks, several unassigned anodic and cathodic peaks are apparent in Fig. 3(A1, A2 and C1–C4). These peaks are caused by the occurrence of side reactions at the titanium substrate, which compete with the formation and oxidation of carbon. As such it is apparent that titanium is not an inert substrate in the molten ternary carbonate system, and so is not an optimal electrode material for current efficient carbon deposition in the system. This finding is consistent with both the conclusions of Song *et al.* based on the stability of titanium wires in LiCl containing molten salts,^32^ and with the low *i*_0_ value reported here for carbon electrodeposition onto titanium substrates.

The reactions contributing to the appearance of peaks not associated with carbon electrodeposition or oxidation in CVs onto titanium substrates were identified based on the known behaviour of titanium electrodes in molten Li_2_CO_3_–Na_2_CO_3_ and Li_2_CO_3_–K_2_CO_3_ electrolytes. In these systems the spontaneous formation of titanium carbides leads to the occurrence of three main side-reactions (eqn (6)–(8)),^52^ the forward and reverse reactions for which contribute to the appearance of the A1–A2 and C1–C4 peaks present in Fig. 3.

6 TiC + 3O^2−^ → TiO + CO_2_ + 2e^−^

7 *z*TiC + Li_2_CO_3_ + (2*z* + *t* − 1)O^2−^ → → Li_2_Ti_*z*_O_*t*_ + (*z* + 1)CO_2_ + 2(2*z* + *t* − 1)e^−^

8 TiO + O^2−^ → TiO_2_ + 2e^−^

where *z* and *t* depend on the stoichiometry of the resultant titanium oxide.

Based on Fig. 2(c), carbon electrodeposition onto both copper and gold substrates also gives rise to multiple peaks beyond those expected from carbon redox reactions. Similar redox peaks have been observed for carbon electrodeposition onto these metals by previous groups,^30^ and have been assigned to a combination of copper oxide formation and gas evolution processes.^30^ This indicates that both copper and gold may be sub-optimal for carbon electrodeposition from a deposition efficiency standpoint, though these changes on these substrates may lead to interesting variation in the properties of the produced carbons, offsetting the disadvantages of these substrates.

The current efficiency is an important experimental and industrial consideration, as such the changes to the current efficiency of carbon electrodeposition with changes in electrolyte and substrate were investigated. The average yield across all samples was slightly greater than 65%, with variation occurring in the yields of individual samples, even those produced under identical conditions, without any major pattern. This indicated that the yield was subject to variation based on how successful it could be harvested from the substrate. Carbon electrodeposition efficiencies have been reported previously to be in the range from 60–100%,^25–27,30^ with the highest yields being obtained under a N_2_ atmosphere. In this report a CO_2_ atmosphere was used. This was judged to be a better option industrially compared to a N_2_ atmosphere as the presence of CO_2_ is necessary to (1) regenerate carbonate ions within the carbonate melt during electrodeposition, and (2) for the net consumption of CO_2_ to form value-added carbon material. In general, the reported average yield of 65% for carbon electrodeposition from molten carbonate salts is superior or equal to that which has been reported for alternative methods of carbon synthesis such as the pyrolysis of lignocellulosic precursors (40–70%).^53^ This indicates that, if the properties of produced carbon materials lend themselves to industrial application, the production of carbon through the reduction of molten carbonate salts could embody an economically viable method of producing carbonaceous materials.

### Morphological characterization

**Fig. 4:**
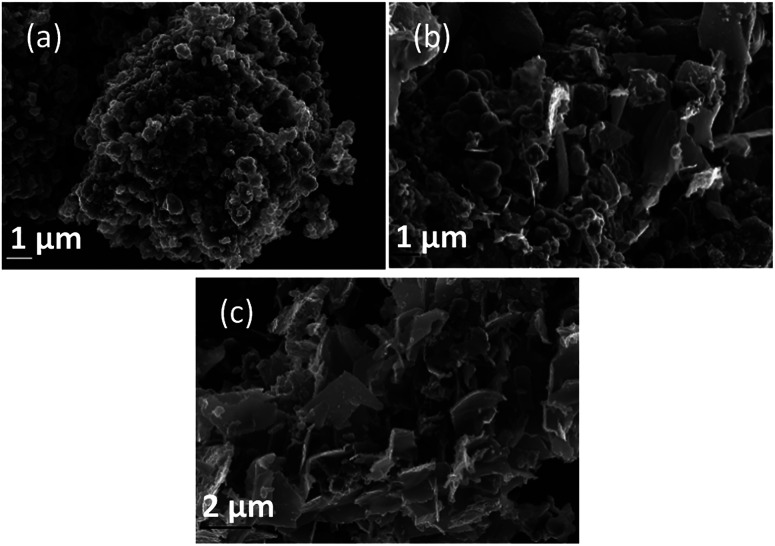
SEM images taken at 20 kV magnification of carbon deposited (a) 500, (b) 600, and (c) 700 °C under otherwise standard conditions.

**Fig. 5:**
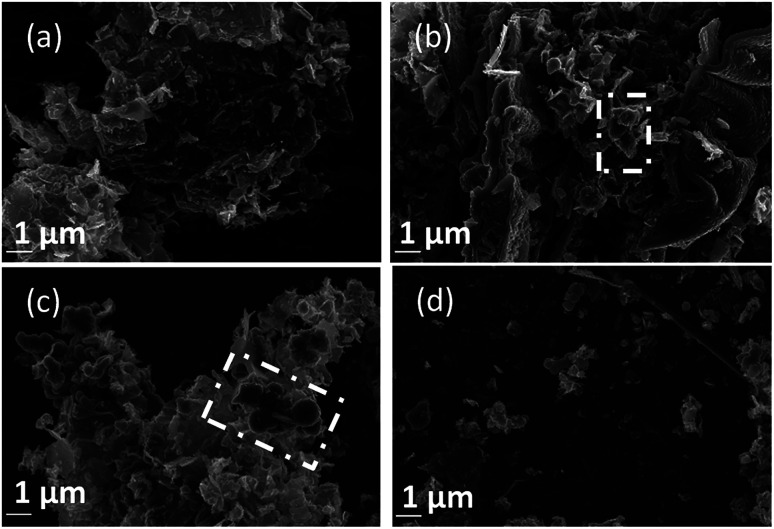
SEM images taken at 20 kV magnification of carbon deposited (a) 0.15, (b) 0.30, (c) 0.60, and (d) 1.20 A cm^−2^ under otherwise standard conditions.

**Fig. 6:**
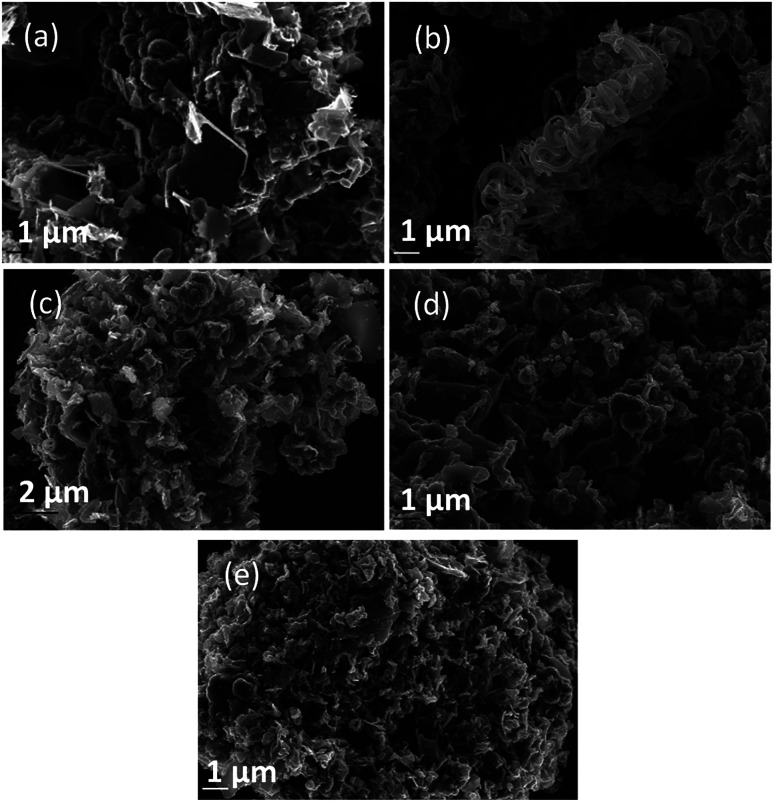
SEM images taken at 20 kV magnification of carbon deposited onto (a) Au, (b) Cu, and (c) Ti; and for carbons deposited in (d) Li–K eutectic and (e) Li–Na eutectics under otherwise standard synthesis conditions.

Morphology was investigated using SEM (Fig. 4–6). The major elements incorporated into the deposited carbons were identified as carbon and oxygen through EDS region scans (ESI Fig. S1†). The morphological features of carbons electrodeposited under different conditions were identified based on both comparison to other synthesized carbons, and through comparison to activated carbon, graphite and conductive carbon black (ESI Fig. S2†).

In general, all the SEM images presented in Fig. 4–6 show a range of morphologies including amorphous phases, plate-like phases similar to those of graphite, spherical aggregates which may be either graphitic or amorphous, and, in some samples, fibre-like structures. Based on Fig. 4(a)–(c), the average size of particles from each phase varies considerably with temperature, with carbons electrodeposited at 500 °C consisting largely of amorphous, folded carbon structures with no tubular aggregates, very few, small, plate-like structures, and with several observable almost-spherical aggregates with an average diameter in the range 230–250 nm.

In comparison, carbon electrodeposited at 600 °C has considerably less amorphous carbon, and a greater proportion of spherical carbon aggregates. Platelet structures similar to those presented for activated carbon in ESI Fig. S2† and some tubular carbon aggregates are also apparent. Spherical aggregates in carbons electrodeposited at 600 °C show surface roughness, indicating they are formed from smaller carbon nanoparticles. The spherical aggregates within carbons electrodeposited at 600 °C largely fall into two size categories, those being small particles with an average diameter in the range 350–400 nm, and larger aggregates with a diameter in the range 800–900 nm. These aggregates are considerably larger than those seen in carbons formed at 500 °C, but the smaller end of the range is comparable to the dimensions of the spherical aggregates apparent in carbons electrodeposited at 700 °C (350–400 nm). This may indicate that when relatively low temperatures are used, there is a slight decrease in the size of spherical carbon aggregates. This may be an effect of the smaller amount of carbon which is electrodeposited in an ordered structural arrangement in carbons electrodeposited at reduced temperatures. Carbons prepared at 600 °C also show a considerable degree of plate-like carbon, which aggregates to form particles 1.5–1.6 μm in size. The lack of this carbon phase at 500 °C indicates that it becomes more energetically favourable for graphitized, plate-like phases to form under these conditions. This is confirmed by SEM images of carbons electrodeposited at 700 °C (Fig. 4(c)), which show no amorphous component, being largely dominated by plate-like structures 1.4–2.0 μm in size. These plates are generally larger than those observed in carbons electrodeposited at 600 °C, however this may be an artefact of the increased number of plates with well-defined end points in carbons electrodeposited at 700 °C. The increasing graphitic character of higher temperature carbons is consistent with both energetic considerations for the systems, and with the earlier mechanistic studies of carbon electrodeposition. From a mechanistic standpoint, as the temperature increases, the overpotential for carbon electrodeposition also increases. This was discussed previously based on the reduced onset potential of carbon electrodeposition in Fig. 2(a), where it was attributed to the increased thermal energy of the system leading to an increase in how energetically favourable it is for carbon in graphitic, sp^2^ hybridized conformation to deposit. This is due to the relative energetics of amorphous, sp^3^ hybridized phases and graphitic, sp^2^ hybridized phases, with the Gibbs energy of formation of amorphous carbon being between that of graphite (0 kJ mol^−1^) and diamond (2.90 kJ mol^−1^).^50^ This indicates that graphitic phases are energetically more stable than amorphous phases so, as the overpotential and thermal energy of carbon electrodeposition is increased, we expect more graphitic carbon formation.

Since the formation of graphitic phases appears to be contingent on the energy of the system and the overpotential of carbon electrodeposition, we expect differences to exist in the graphitic character of carbons electrodeposited at both varied current densities (as this will directly influence the overpotential of carbon electrodeposition) and when depositing onto different substrates (which exhibit varied electrodeposition onset potentials). This expected trend of increased graphitic character in carbons electrodeposited at elevated current densities has been demonstrated experimentally. Based on Fig. 5(a)–(d) and ESI Fig. S3,† as the current density of carbon electrodeposition increases between 0.15–1.20 A cm^−2^, a considerable increase in the graphitic character of the electrodeposited carbon is observed. This effect is most noticeable when comparing carbon electrodeposition at 0.15 A cm^−2^ (Fig. 5(a)) and 1.20 A cm^−2^ (Fig. 5(d)). Carbon electrodeposition at both 0.30 A cm^−2^ and 0.60 A cm^−2^ produces carbons with similar morphologies, with both plate-like and spherical morphologies being apparent. The radius of spherical phases in carbons electrodeposited at 0.30 A cm^−2^ (annotated in Fig. 5(b), ∼300 nm) tends to be smaller than those present in carbons electrodeposited at 0.60 A cm^−2^ (annotated in Fig. 5(c), ∼600 nm). Based on previous analysis of the effect of increased overpotential on the morphology of the electrodeposited carbon, this may indicate that these carbon aggregates consist of graphitized carbon.

Based on Fig. 6(a)–(c), as is expected based on previous analysis, the substrate onto which carbon electrodeposition occurs leads to considerable variation in carbon morphology. This effect is noticeable particularly when comparing the morphology of carbons electrodeposited onto either Au or Ti substrates to those electrodeposited onto Cu (Fig. 6(b)), which show considerable degrees of folded, fibre-like phases absent when using other substrates. The mechanism of formation and reasons for the formation of these fibre phases on Cu is currently under investigation. No major unique features are present in the SEM images presented for carbons electrodeposited onto Au (Fig. 6(a)) as compared to Ti (Fig. 6(c)), except for a slight difference in the size of the graphitic domains. This may be due to the large range of competing cathodic reaction previously observed when electrodepositing carbon from molten carbonates onto Ti, which could lead to a lowered fraction of carbon forming with sufficient energy to obtain a graphitic composition, instead depositing as amorphous carbon, so small platelets of graphitic carbon may be preferentially formed in this case. In general, carbon electrodeposited onto Au appears to consist of large, quasi-spherical aggregates in the order of 1.0–1.5 μm, flake-like structures with lengths of 1.0–2.0 μm, and randomly located amorphous phases. Compared to this, carbon electrodeposited onto Ti consists largely of small, flake-like aggregates with sizes of 0.6–1.0 μm, along with amorphous phases. The morphology of carbon electrodeposited onto Cu consists of fibre structures with diameters of up to 400 nm, plate-like phases, and amorphous phases. The fibre-like structures consist of thick, hollow tube structures (ESI Fig. S4†). Both wide hollows of up to 300 nm and, more frequently, thin tube openings in the order of 40 nm were present. This wide variation of morphology depending on substrate indicates that this variable plays a significant role in carbon electrodeposition.

From a comparison of Fig. 4(b), a typical SEM image of carbon electrodeposited in ternary carbonate eutectic, to Fig. 6(d) and (e), SEM images of carbons electrodeposited in binary carbonate systems, it is apparent that there are no major changes in the carbon phases present upon electrolyte variation. In spite of this, the surface folding and apparent porosity of carbon electrodeposited in binary Li–Na eutectics (Fig. 6(e)) is considerably lower than that of either carbon electrodeposited in either a binary Li–K eutectic (Fig. 6(d)) or a ternary Li–Na–K eutectic (Fig. 4(b)). This was confirmed through measurement of the BET SSA for carbons produced in Li–K and Li–Na–K systems (322 m^2^ g^−1^ and 327 m^2^ g^−1^, respectively) as compared to those of carbon produced from a molten Li–Na system (188 m^2^ g^−1^). From this it is apparent that carbon electrodeposition from both Li–K and Li–K–Na eutectics leads to high surface area materials.

In each case the BET SSAs of the carbonate-derived carbon materials was considerably lower than those reported for many current high performance carbon materials, such as activated carbons or sulphur-doped activated carbons which show SAs of upwards of 3000 m^2^ g^−1^.^54^ However, due to the high degree of oxygen functionalization hinted at in the examined materials but EDS spectra such as that shown in ESI Fig. S1,† it is likely that the electrochemical performance of the examined carbonate-derived carbons will be higher than would be predicted based on simple surface area considerations.

In general, reduced amorphous character and elevated graphitic character tends to be observed when electrodeposition is carried out at both increased temperatures and current densities. As several major applications of amorphous carbons, such as their use as chemical adsorbents and in electrochemical capacitors, are positively influenced by elevated surface areas (or electroactive surface areas)^55^ it can be predicted that the optimal conditions for producing carbons for these applications from molten carbonate systems will be with low temperature, low current density electrodeposition. This is highly desirable as it means that low energy input is predicted to be optimal for the synthesis of practical materials.

Carbon morphology was further investigated using TEM. TEM has been used previously to characterize the manner in which carbon structure varies with electrodeposition conditions.^39^ The trends identified during these studies are consistent with the trends identified here based on SEM studies, with TEM imaging (ESI Fig. S5†), largely confirming variation in morphological features to be due to structural variation in the electrodeposited carbons. Due to the largely amorphous nature of electrodeposited carbons, many show little to distinguish them from the carbon-coated copper grid used during TEM imaging, however carbon electrodeposited between 500 °C and 700 °C clearly shows the expected major phases with changes in electrodeposition conditions. At 500 °C (Fig. S5(a) and (b)†) there is little distinction between the synthesized carbon and the amorphous carbon coating of the Cu grid used during TEM. Small segments of ordered carbon are largely confined to the edges of particles (an example is annotated in Fig. S5(b)†). Based on their ordered nature, these segments likely correspond to graphitic carbon.^56^ The fact that these graphitic phases are observed close to the particle edges may indeed be an edge effect, which leads to a low contrast between carbon phases at the centre of carbon particles compared to that seen towards the edge, or, it may be that these graphitic carbons form relatively fragile shearing points in carbon macroparticles, which are severed during ultrasonication, leaving graphitic carbons concentrated at the edges of particles. If the concentration of graphitic fragments at the edge of particles is due to shearing effects, this may be accounted for by the weak bonding strength of poorly aligned, sp^2^ hybridized graphitic phases compared to amorphous phases, which show a blend of sp^2^ and sp^3^ hybridized carbons.^57^ As the bond strength of sp^3^ hybridized carbon tends to be greater than that of sp^2^ hybridized carbon^58^ weak shear points in the deposited carbons likely consist of sites with elevated graphitic character.

TEM images of carbon electrodeposited at 600 °C (Fig. S5(c) and (d)†) show similar characteristics to those of carbon electrodeposited at 500 °C, however ordered phases are apparent towards the centre of particles rather than solely being present towards the edge (Fig. S5(c); † annotated regions). These graphitic phases are randomly aligned and tend not to show any consistent layer separation distance. The existence of more graphitic phases in carbons electrodeposited at 600 °C, as compared to 500 °C, indicates that elevated temperature contributes to the formation of more graphitized carbon, which is consistent with the results presented previously based on SEM. TEM imaging of carbon electrodeposited at 700 °C (Fig. S5(e) and (f)†) shows high levels of graphitized phases with extended ordering and alignment. Variation in the orientation of the graphitized phases is apparent in these materials (annotated in Fig. S5(e) and (f)† using the direction of the boxed regions), however the separation of layers is consistently ∼0.35 nm. The diffraction patterns for domains containing graphitic carbon (Fig. S5(g)†) show distorted circles corresponding to the overlapping of many similar crystal diffraction patterns aligned in different directions, leading to multiple light points that join to form a circular region rather than six well-defined light points that would normally be associated with a hexagonal carbon system. These crystals are the multidirectional graphite phases present in carbon electrodeposited at 700 °C. Thus, in terms of morphology, carbon electrodeposited at 700 °C is similar to poorly ordered graphitized carbon with amorphous regions also being apparent.

### Carbon functionalization and bonding

**Fig. 7:**
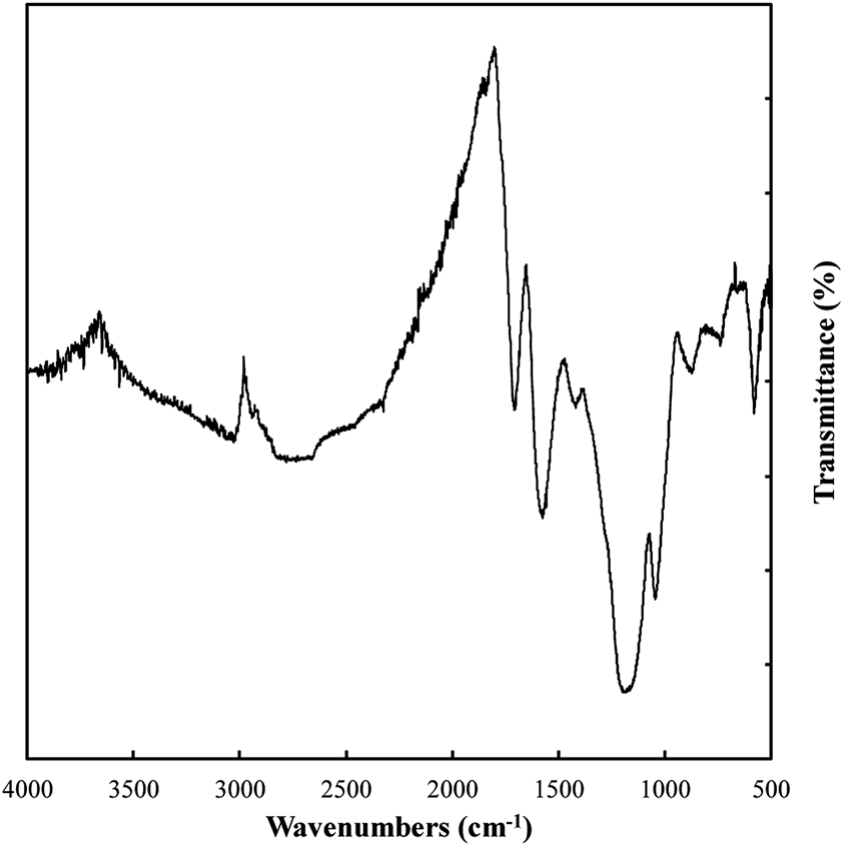
The FTIR pattern of carbon electrodeposited at 500 °C using otherwise standard conditions.

Variations in the chemical bonding and functionalization of carbons electrodeposited from molten carbonate electrolytes were investigated based on FTIR, Raman spectroscopy, XRD, and XPS. FTIR patterns for these materials are relatively similar, with the only major difference being peak intensity. Due to these similarities the spectrum with the most intense peaks (that for a carbon electrodeposited at 500 °C, Fig. 7) was taken to exemplify the spectra of all materials. The bonding interactions present in the electrodeposited carbons were identified as shown in Table 3.

**Table 3:** Typical FTIR vibrations of carbons electrodeposited from molten carbonate electrolytes. Wavenumbers marked by * represent tentative assignment of peaks where the magnitude of the peak is not much larger than the signal noise

Observed wavenumber (cm^−1^)	Signal origin^59,60^
2980	C–H_3_
2925	–CH_2_−
2660	C–H (aldehyde)
2320–2000*	C≡C
2000–1900	C=C=C
1835	OC=OO
1720–1650	C=O, C=C
1580	CC
1410	Alkane/alkene C–H
1140	C–C
1030	C–H/C–O
850	Alkene C–H
570	CC–H

Based on Table 3, carbons electrodeposited from molten carbonates show the presence of a range of C–H, C–C, and C–O environments, with C–H peaks characteristic of both sp^3^ and sp^2^ hybridized carbon being apparent. In addition, C–C peaks that relate to sp^3^, sp^2^ and, to a very small degree, sp hybridized carbon are also present. Finally, both C–O and C=O bonding is observed. These structural characteristics indicate that these carbons take the form of combined C–C and C=C bonded carbons with oxygen functionalized surfaces. The presence of graphitic phases is in keeping with the previous SEM and TEM results indicating the carbons consist of mixed amorphous, sp^3^ (non-graphitic) carbons and graphitic, sp^2^ hybridized carbon.

**Fig. 8:**
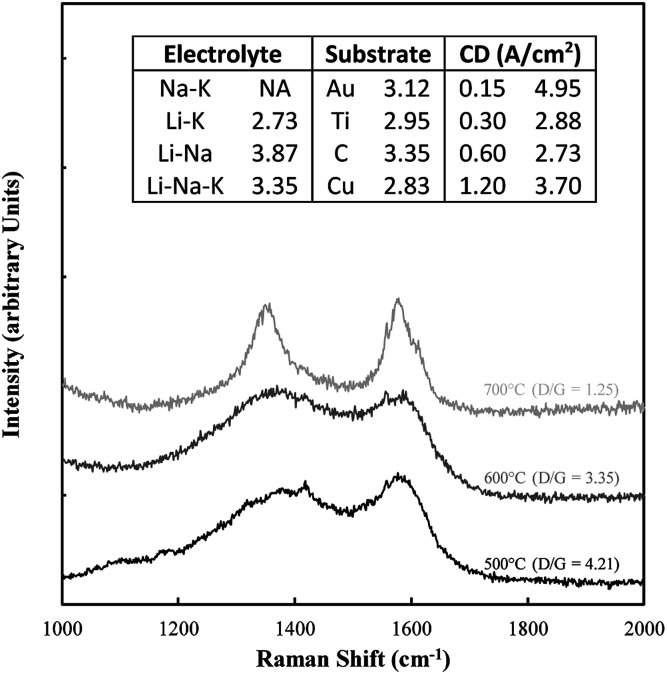
Example of Raman data collected on the electrodeposited carbon samples. Also included are the corresponding D/G ratios.

Typical examples of the Raman data collected on these samples are shown in Fig. 8. The spectral region considered (1000–2000 cm^−1^) contains the D and G bands for carbons, in which case there is considerable overlap. Also shown in this figure is the D/G ratio for all samples. In all cases this ratio was well above 1 indicating a substantially disordered carbon, consistent with the morphological analysis. The most ideal sequence is for changing temperatures, in which case an increase in temperature clearly graphitized the material, leading to a lower ratio.

**Fig. 9:**
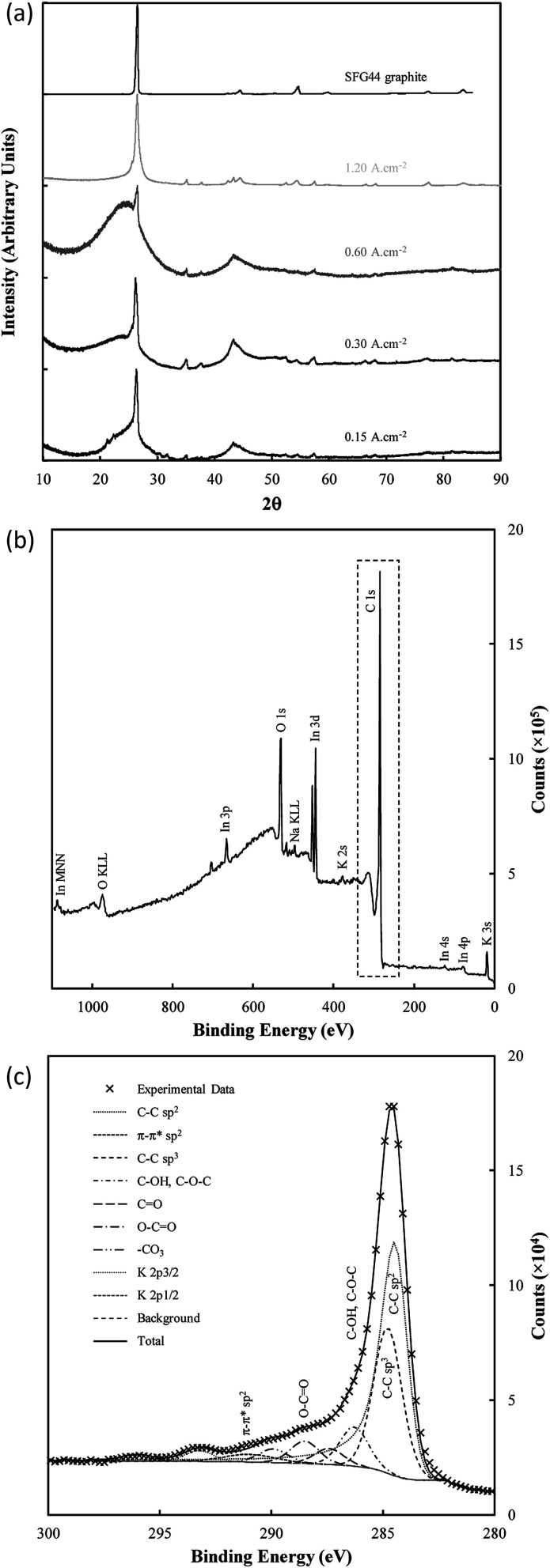
(a) XRD patterns of SFG44 graphite and carbon electrodeposited with different current densities under otherwise standard conditions; (b) complete XPS spectra of standard electrodeposited carbon mounted onto indium foil, and (c) the corresponding C 1s region.

The structure of electrodeposited carbon was further investigated using XRD (Fig. 9(a)). Structural changes in electrodeposited carbons have been investigated previously by this group using XRD.^39^ The major features of the XRD patterns are exemplified in Fig. 9(a), with the patterns showing the presence of amorphous and graphitic carbon phases through the very broad peak centred at 23° 2*θ* (low order carbon) and through the peaks at 26.5° 2*θ* and 44.5° 2*θ* corresponding to the 002 and 101 planes of hexagonal graphitic carbon.^61^ In addition, carbons electrodeposited at high current densities (1.2 A cm^−2^), show peaks characteristic of lithium carbide (25°, 38°, 51° and 82° 2*θ*).^62^ These peaks are also evident, although at a lower intensity, when electrodepositing at lower current densities (0.3 A cm^−2^). The formation of lithium carbide is of course undesirable, reducing the total carbon yield towards the 65% yield noted previously. The presence of lithium carbide is likely the result of the highly negative deposition potential necessary to achieve current densities of 1.2 A cm^−2^, which was in the order of −3 to −4 V *vs.* C/CO_2_/CO_3_^2−^ for much of the early carbon electrodeposition period.

The graphitic XRD peaks were used to calculate the unit cell dimensions and *d*-spacings for the electrodeposited carbons through eqn (10)–(12); *i.e.*,

10 *λ* = 2*d*_*hkl*_ sin(*θ*)

11 *a* = *b* ≠ *c*

12
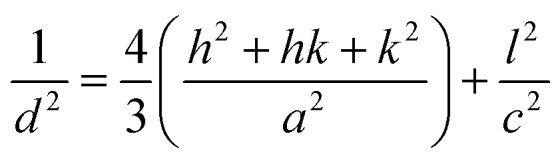
where *h*, *k* and *l* are Miller indices, *λ* is the wavelength of the Cu K-α radiation (1.508 Å), *d* is the spacing for the lattice plane specified by the associated Miller indices (Å), and where *a*, *b*, and *c* are axis lengths (Å). Table 4^39^ shows the lattice parameters and unit cell volumes of carbons electrodeposited under a range of conditions from molten carbonate salts. The carbons are labelled using identifiers derived from the variable used to produce them, with other synthesis conditions being otherwise the standard conditions. Carbons which do not show peaks characteristic of graphitized carbon have their interlayer spacings and lattice parameters listed as N/A.

**Table 4:** The interlayer plane spacing, lattice parameters and unit cell volumes of the graphitised phases of synthesised carbons produced by varying singular parameters of the standard conditions during carbon synthesis. Parameters were varied from the specified categories of deposition temperature, current density, carbonate eutectic used, or substrates used during carbon synthesis

	Sample	*d*_002_ (Å)	*d*_101_ (Å)	*a* (Å)	*c* (Å)	Volume (Å^3^)
Control samples	Graphite	3.36	2.03	2.46	6.71	35.33
	Activated carbon	N/A	N/A	N/A	N/A	N/A
Temperature (°C)	500	N/A	N/A	N/A	N/A	N/A
	600	3.38	2.06	2.50	6.77	36.70
	700	3.37	2.04	2.47	6.73	35.69
Current density (A cm^−2^)	0.09	3.38	2.05	2.48	6.77	35.96
	0.15	3.39	2.06	2.49	6.78	36.53
	0.30	3.38	2.06	2.50	6.77	36.70
	0.60	3.37	2.05	2.48	6.74	35.90
	1.20	3.36	2.04	2.47	6.73	35.52
Carbonate eutectic used	Li/K	3.36	2.05	2.48	6.73	35.84
	Li/Na	3.36	2.06	2.49	6.73	36.18
	Li/K/Na	3.36	2.04	2.48	6.72	35.70
Substrate used	Graphite	3.36	2.04	2.48	6.72	35.70
	Gold	3.42	2.06	2.49	6.85	36.80
	Copper	3.35	2.05	2.48	6.71	35.72
	Titanium	3.37	2.05	2.48	6.74	35.86

There are only small variations in the unit cell dimensions of the electrodeposited carbons with changes to synthesis conditions. Carbons electrodeposited at both elevated temperatures and at elevated current densities tend to have graphitized phases with unit cell dimensions similar to that of standard graphite compared to carbon electrodeposited at lower temperatures and current densities. This indicates that the elevated quantity of graphite-like phases in carbons electrodeposited at higher temperatures and current densities is also structurally more similar to graphite than similar phases in carbons formed under low temperatures or at low current densities. This effect holds more strictly for carbons electrodeposited at different temperatures than it does for carbons electrodeposited at different current densities which tend to show little change in the unit cell structure of the graphitized phase. For electrodeposition at 500 °C there is no graphitized carbon apparent. This is in direct conflict with the results of FTIR, and either indicates that the relative magnitude of the amorphous phase signal to the graphitic peak in these carbons is too large for the graphitic peak to be apparent (due to the low amount of graphitic carbon), or that an element of preferred particle orientation is present in these amorphous materials. Carbons electrodeposited at 500 °C showed XRD patterns with a fair degree of similarity to examined activated carbons, which were near-amorphous. Conversely, carbons deposited at both 700 °C and at 1.20 A cm^−2^ showed little or no signal relating to amorphous carbon. This indicates that, at high temperatures and at very high current densities, the graphitized portion of synthesized carbons is considerably greater than the near-amorphous portion, which is consistent with our previous findings.

Very little variation was observed in the interlayer spacing of synthesised carbons with changes to the electrolyte, with each examined subset of carbons showing an average *d*_002_ spacing of 3.36 Å, consistent with the *d*_002_ spacing of examined graphite. There was, however, sight variation present in the calculated *d*_101_ spacing with changes to the electrolyte, with the lowest average *d*_101_ spacing of 2.04 Å being obtained when the ternary carbonate eutectic was used. The largest *d*_101_ spacing of 2.06 Å was obtained when using the Li/Na binary eutectic. Compared to this small degree of variation, the electrodeposition substrate appears to have a considerable effect on the graphitic lattice parameters, with carbons electrodeposited onto graphite and Cu substrates showing unit cell parameters and interlayer spacings most similar to graphite. Carbon electrodeposited onto Au had a unit cell considerably larger than that of graphite. This may make carbons deposited in this manner more suitable for battery applications than other carbons, as it may increase the ability of the material to intercalate lithium ions between the graphite sheets.

**Fig. 10:**
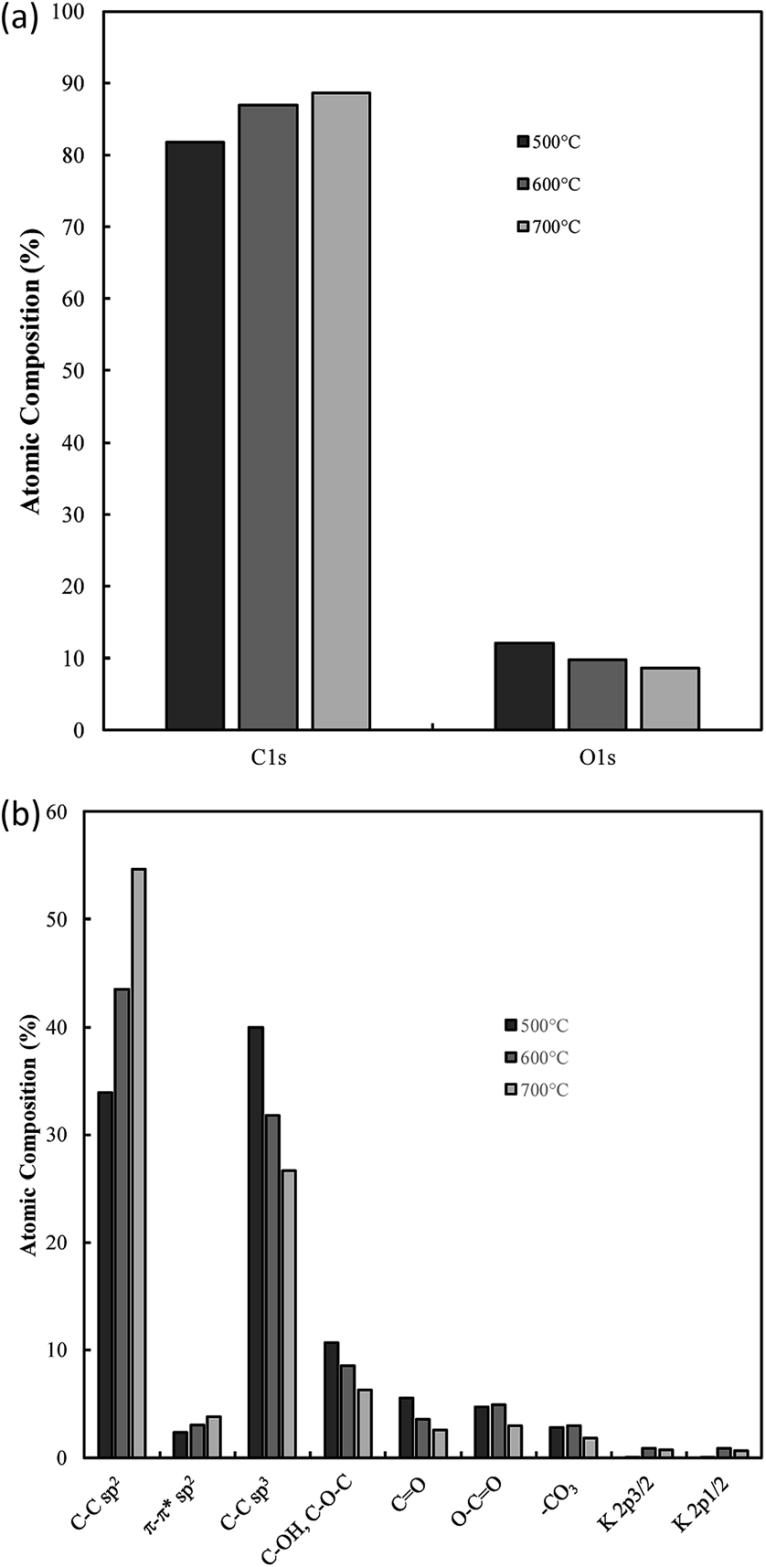
(a) Atomic composition of functionalized carbon materials synthesised at 500 °C, 600 °C and 700 °C attributed to the C 1s and O 1s peaks of the XPS survey spectra and (b) the % atomic composition of carbon in different bonding environments in carbon materials synthesised at 500 °C, 600 °C and 700 °C as calculated from C 1s region scan peak fitting.

Bonding and functionalization in the examined carbon materials was investigated further through XPS (Fig. 9(b) and (c)). The XPS survey scan and C 1s region scan of raw indium foil (ESI Fig. S6(a and b)†) was used to give an idea as to the form of adventitious and contaminant carbon present during the XPS of synthesised carbons. It was found that the foil showed a combination of indium, carbon and oxygen peaks typical of metallic foils, and that potential peaks relating to further metallic impurities were well below 0.25% of the composition of the foil. Based on survey scans (Fig. 9(b)), carbons formed through the electrolytic reduction of molten carbonate salts show major peaks corresponding to carbon, oxygen and indium, with small peaks corresponding to the presence of sodium and potassium being present as well. These trace peaks result from the presence of solidified carbonates sequestered in the pores of the examined carbon materials. Accounting for the carbon and oxygen contributions of the exposed indium surface, no trends were observed in the concentrations of impurities and trace elements in the examined carbon materials, however major trends were observed in the carbon and oxygen contents of electrodeposited carbons. These trends were investigated for carbons electrodeposited at temperatures between 500–700 °C, which showed well-defined, systematic morphological changes (Fig. 10).

From Fig. 10(a) it is apparent that the carbon content increases in carbons with and increased electrodeposition temperature, varying between 81 at% at 500 °C to 88 at% at 700 °C. Counter to this, the oxygen content decreases, varying from 14 at% in carbons electrodeposited at 500 °C to 9 at% when 700 °C was used. This indicates that the oxygen functionalisation tends to decrease with elevated electrodeposition temperature. This inverse relationship likely contributes to previous findings which indicated that the pseudo-capacitive performance of carbons was inversely related to electrodeposition temperature,^39^ and is likely related to increased oxygen functionalization being present in more amorphous carbons formed during molten carbonate reduction.

Region scans of the C 1s and O 1s binding energy domains were used to evaluate the relative concentrations of major carbon bonding environments present in the synthesised materials. Due to the oxygen-rich nature of the indium foil used to mount carbons and the broad nature of the O 1s peak no major unique results could be derived in the region. As such the major focus in this section is dedicated to the fitting assigned to the C 1s peak (Fig. 9(c)). Due to the number of peaks apparent in the high binding energy region of these scans the exact composition of minor peaks in this region is subject to variability based on starting conditions of the fitting. As such relative variations are stressed here over exact composition values. In general, synthesized carbons show amorphous (sp^3^) C–C bonds, sp^2^ C=C bonds (and an associated π–π* shake-up peak), C–O–C, C=O, and O–C=O bonding, and peaks associated with carbonate/potassium. C–OH peaks share similar binding energy to C–O–C peaks so may also be present but, based on the earlier results of FTIR studies, this is unlikely to be the case. In general, XPS identifies many of the groups and bonding environments theorized to be present based on FTIR studies, lending validity to the results of both forms of analysis. The binding energies used to fit the C 1s regions for carbons electrodeposited at 500–700 °C are supplied in ESI Table S1.† Variation in the relative composition of electrodeposited carbons with synthesis temperature, which was taken to be a well-defined series to model the influence of changes in ordered character on the bonding of synthesized carbons, is shown in Fig. 10(b). In general, as the temperature increases the graphitic sp^2^ character of carbon increases markedly, with carbon electrodeposited at 500 °C showing close to 30 at% sp^2^ carbon, while that electrodeposited at 700 °C showing around 50 at%. This trend is internally consistent with that shown in the π–π* bonding of the materials and is inverse to that observed in the variation in sp^3^ structure of carbons with electrodeposition temperature, with amorphous character tending to be considerably higher in carbons electrodeposited at 500 °C than in those at 700 °C. This agrees with previous SEM, TEM and XRD results, all of which found an increase in graphitic character with increasing temperature. This indicates that by controlling the electrodeposition temperature, it will be possible to create materials with elevated graphitic character. Materials of this sort are expected to perform well in applications such as battery systems, which utilise lithium ion insertion into a graphitic structure for long-term energy storage and conversion. Conversely, lowering the electrodeposition temperature leads to amorphous structures suitable for capacitive applications. In terms of oxygen functionalization, low levels of oxygen functional groups are observed in all samples, with the total functionalization contributing to less than a 20 at% of the total composition. Excepting O–C=O bonding, which, based on Fig. 10(b) shows similar amounts in carbons electrodeposited at 500 °C and 600 °C, the concentration of C–O–C and C=O bonding in carbons tends to decrease with elevated temperatures. Since carbons which show elevated oxygen functionalization have been previously shown to demonstrate elevated pseudo-capacitive performance,^39^ it is clear that this will have a beneficial effect. In addition, XPS region scans of the C 1s region allow for examination of the most frequently present functional groups in carbon materials. In the case of carbon electrodeposited at low temperatures, the greatest degrees of functionalization are by C–O–C groups and C=O groups. The presence of these electronegative groups may be predicted to lead to considerable bond bending at their location, which is, in turn, predicted to lead to a considerable increase in the amorphous, disordered structure of sp^3^ phases and a decrease in the graphitic character of surrounding sp^2^ carbon phases. This is consistent with the previous XRD results, which showed a decrease in the deviation of the graphitic unit cell from ideal behaviour with increased electrodeposition temperatures, and with the results of TEM and SEM, showed the amorphous character of carbons to decrease and the graphitic character of carbons to increase with elevated temperatures. The presence of O–C=O groups can be attributed to dangling carbon chains present in the synthesized carbon materials. It is likely, due to the highly polar nature of these groups that they will contribute substantially to pseudo-capacitance of these carbon materials. As such their electrochemical performance in electrochemical capacitor applications is expected to be at its maximum in highly amorphous carbons, which may be synthesized with a low overpotential, such as with low temperature or current density. The presence of carbonate and K groups in the examined materials is likely due to sequestration of these groups in the porous structure of carbons during growth.

### Electrochemical performance

**Fig. 11:**
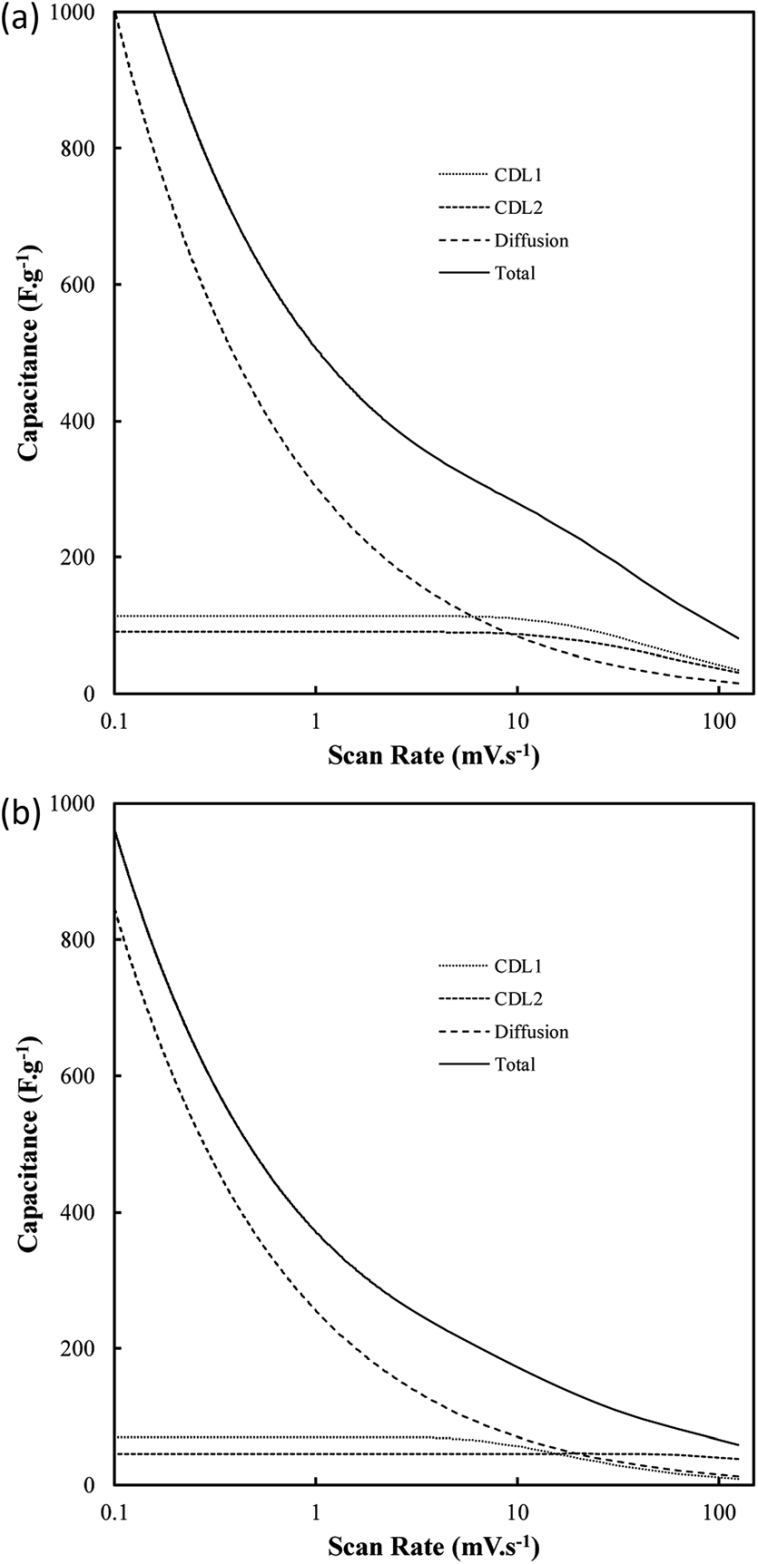
The influence of scan rate on the contributions to the total capacitance of (a) carbon deposited at 0.15 A cm^−2^ and otherwise standard conditions, and (b) activated carbon. *C*_D_ represents capacitance due to diffusion, while *C*_DL1_ and *C*_DL2_ represent the double layers associated with the nano and microporous surface area and the external carbon surface area respectively.

One of the most interesting potential applications of functionalized carbon materials is in energy storage systems such as electrochemical capacitors. The performance of electrodeposited carbons has been studied previously using cyclic voltammetry (CV) and step potential electrochemical spectroscopy (SPECS).^28^ It has been shown previously using CV (ESI Fig. S7†) that these carbons have specific capacitances as high as 230 F g^−1^ in aqueous (0.5 M H_2_SO_4_) media when electrodeposited at low current densities (0.15 A cm^−2^). Likewise, high capacitive performance was also found for carbons electrodeposited at low temperatures (500 °C), the use of a graphite substrate, and a ternary or Li–K eutectic. The best of these capacitance values compares favourably to the performance of commercially available activated carbon,^63,64^ as well as to the performance of experimentally tested activated carbon prepared through the pyrolysis and subsequent chemical activation of coconut husks (220 F g^−1^). It is apparent that superior capacitive performance is obtained in carbons with low graphitization, high amorphous character, and high functionalization. This is consistent with an intuitive interpretation of double layer capacitances, where it is expected that more folded, porous and amorphous carbons should have greater surface areas accessible for the formation of electric double layers. Beyond this, it is likely that a major contributor to the high capacitive performance of these materials is the high degree of oxygen functionalization present in these materials. This conclusion is consistent with previous findings obtained from deconvolution of the double layer and pseudo-capacitive contributions to the total performance.^28,39^ It was found, based on an analysis such as shown in Fig. 11(a) and (b), that the pseudo-capacitive contribution to the overall performance of carbonate-derived carbons (*C*_D_) was extremely high at slow scan rates, but decayed considerably as the scan rate increased due to kinetic limitations. This decay is less evident in terms of double-layer capacitance due to the rapid kinetics of double layer formation. In most cases *C*_DL1_ (double layer capacitance associated with porous surface area) tends to be more affected by increasing scan rates than *C*_DL2_ (geometric surface area), which is due to *C*_DL1_ being dependent upon electrolyte access to the microporous surface area of the examined carbons, which is hindered at high scan rates. Maximum low rate capacitive performance was obtained in carbon electrodeposited at low current densities (0.15 A cm^−2^), with capacitances of as high as 400 F g^−1^ being achieved at 10 mV s^−1^.^39^ These values, in keeping with the results of CV studies, were greater than those achieved for other amorphous, low graphitisation systems such in carbon electrodeposited at 500 °C (250–300 F g^−1^), and were comparable to the maximum performances reported for other new, high performing supercapacitive materials such as N, P co-doped activated carbons.^65^ Each of these amorphous, highly functionalized systems performed considerably better than their graphitized, low functionalized counterparts, with carbon electrodeposited at 1.2 A cm^−2^ and 700 °C showing capacitances in the range 100–150 F g^−1^ at 10 mV s^−1^ scan rates (ESI Fig. S8†). As such, it is apparent from the previous physical, morphological and structural studies, that optimal capacitive performance is obtained from carbons with high oxygen functionality, high amorphous and low graphitic structure, and highly folded, apparently porous morphologies. From previous sections it is evident that this arises from carbons produced at low temperatures and current densities using a graphite substrate (which also minimises the presence of impurities but inhibits the formation of unique carbon morphologies such as carbon nanotubes), in a molten ternary Li–Na–K or binary Li–K eutectic. Due to the high level of influence that temperature variation has been shown to have on these carbon morphologies, and due to the reduced melting point of the ternary compared to Li–K binary eutectic (397 °C compared to 489 °C),^40,41^ it is recommended that, for the synthesis of optimized supercapacitor carbons from molten carbonate salts, carbon electrodeposition at low temperature and low current density be allowed to proceed at a graphite working electrode in a ternary Li–Na–K carbonate melt.

## Conclusions

The mechanistic specifics of the electrodeposition of carbon from molten carbonate salts have been investigated through CV and Tafel analysis. A secondary mechanism of carbon electrodeposition from molten carbonates based on the presence of pyrocarbonate ions in the molten media has been presented and is theorized to occur in the presence of a CO_2_ atmosphere. Variation in the potential of carbon electrodeposition from molten carbonate salts has been attributed largely to variation in the internal energy of the systems and to changes in the potential of electrodeposition from molten carbonate systems when depositing onto different substrates. Tafel kinetics has been investigated and charge transfer coefficients (and associated exchange current densities) have been determined, with it being shown that the systems behave in considerably different ways to ideal reversible systems. The presence of side reaction on Au, Cu, and Ti substrates have been identified, with these reactions having a generally negative effect on the suitability of these metals for use as substrates. In spite of this it has been found that carbon electrodeposition onto Cu can lead to the formation of nanofiber and nanotube morphologies not seen when electrodepositing onto other substrates.

Morphological studies based on SEM and TEM have shown that carbons consist of varying degrees of folded, amorphous and graphitic structures, with graphitic character tending to increase with increased current densities and temperatures of carbon electrodeposition.

Functionalization and bonding within the carbons has been investigated, and the materials have been shown to consist of sp^2^ and sp^3^ hybridised carbons with C–C, C–H, C=O, C–O–C, C=O, and O–C=O chemical environments, with both sp^3^ structure and oxygen functionalization of carbons being at their highest under conditions that produce carbons with the most disordered morphologies. The unit cell volumes of graphitic phases in electrodeposited carbons have been investigated, and it has been shown that those carbons with the lowest amorphous character show *d*_002_ and *d*_101_ spacings with the greatest similarity to graphite.

The capacitive performance of synthesized carbons has been evaluated, and maximum performances of 400 F g^−1^ at 10 mV s^−1^ have been obtained. As such it is concluded that the reduction of molten carbonate salts embodies an interesting method of producing capacitive carbons with physical characteristics that are dependent on the synthesis conditions for the materials.

## Conflicts of interest

There are no conflicts to declare.

## Supplementary Material

RA-009-C9RA05170H-s001
