# Supplementary material for: Physical characteristics of capacitive carbons derived from the electrolytic reduction of alkali metal carbonate molten salts
Source: RSC Adv. 2019 Nov 11;9(63):36771–87. doi: 10.1039/c9ra05170h (PMC9075116; doi:10.1039/c9ra05170h)
Supplement: RA-009-C9RA05170H-s001 [file RA-009-C9RA05170H-s001.pdf]

Electronic Supplementary Material (ESI) for RSC Advances.
This journal is © The Royal Society of Chemistry 2019

Table S1. Compositions derived from comparisons between the collected C1s region scans of carbons formed through the electrolytic reduction of molten carbonate salts at 500°C, 600°C and

Sample Identifier	Name	Position	FWHM	%At Conc
NMN053 S2: 500-T	SP2 C-C	284.5	1.3	33.9
	Pi-Pi* (SP2)	291.0	2.7	2.3
	SP3 C-C	284.8	1.5	40.0
	C-OH, C-O-C	286.2	1.5	10.7
	C=O	287.2	1.5	5.5
	O-C=O	288.5	1.5	4.7
	Carbonate	289.7	1.5	2.8
	K 2p3/2	293.1	1.5	0.1
	K 2p1/2	295.9	1.5	0.1
NMN053 S3: 600-T	SP2 C-C	284.5	1.3	43.5
	Pi-Pi* (SP2)	291.0	2.7	3.0
	SP3 C-C	284.8	1.5	31.8
	C-OH, C-O-C	286.3	1.5	8.6
	C=O	287.4	1.5	3.5
	O-C=O	288.5	1.5	4.9
	Carbonate	289.9	1.5	3.0
	K 2p3/2	293.2	1.5	0.9
	K 2p1/2	296.0	1.5	0.9
NMN053 S4: 700-T	SP2 C-C	284.5	1.3	54.7
	Pi-Pi* (SP2)	291.0	2.7	3.8
	SP3 C-C	284.8	1.5	26.6
	C-OH, C-O-C	286.3	1.5	6.3
	C=O	287.3	1.5	2.5
	O-C=O	288.5	1.5	2.9
	Carbonate	289.8	1.5	1.8
	K 2p3/2	293.4	1.5	0.7
	K 2p1/2	296.2	1.5	0.7

700°C.

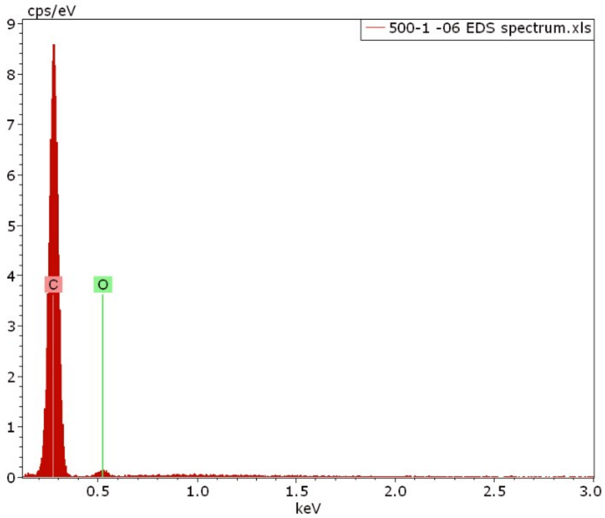

Figure S1. EDS region scans for (a) carbon deposited at 500°C under otherwise standard conditions and (b) carbon deposited onto iron under otherwise standard conditions.

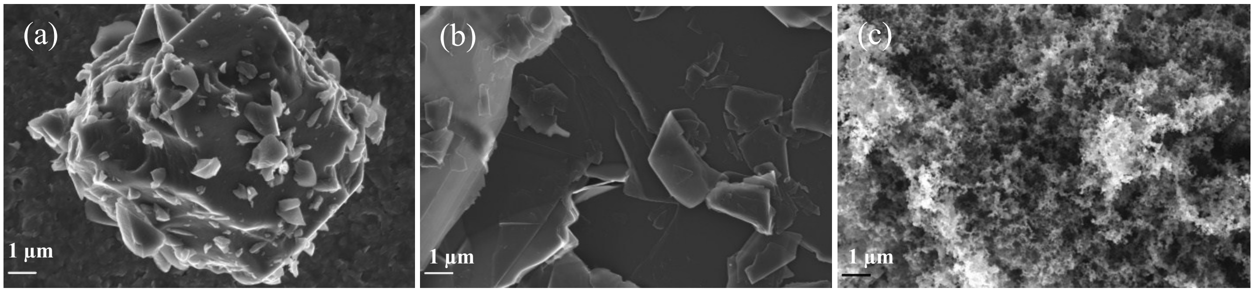

Figure S2. SEM images showing typical morphological features of (a) activated carbon, (b) graphite, and (c) conductive carbon black. All images were taken at 20 kx magnification.

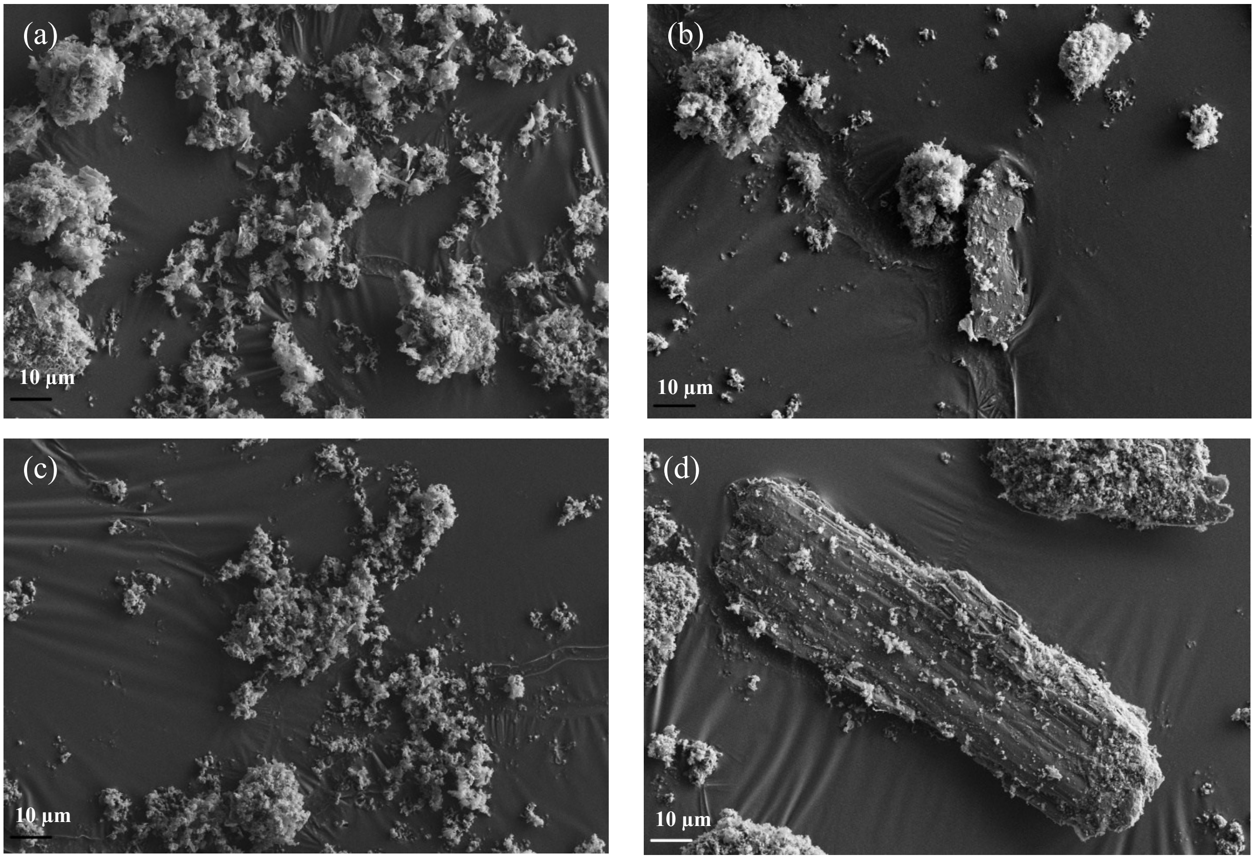

Figure S3. SEM images of carbons deposited at (a) 0.15 $A.cm^{-2}$, (b) 0.30 $A.cm^{-2}$, (c) 0.60 $A.cm^{-2}$ and (d) 1.20 $A.cm^{-2}$ as taken at 2 kx magnification.

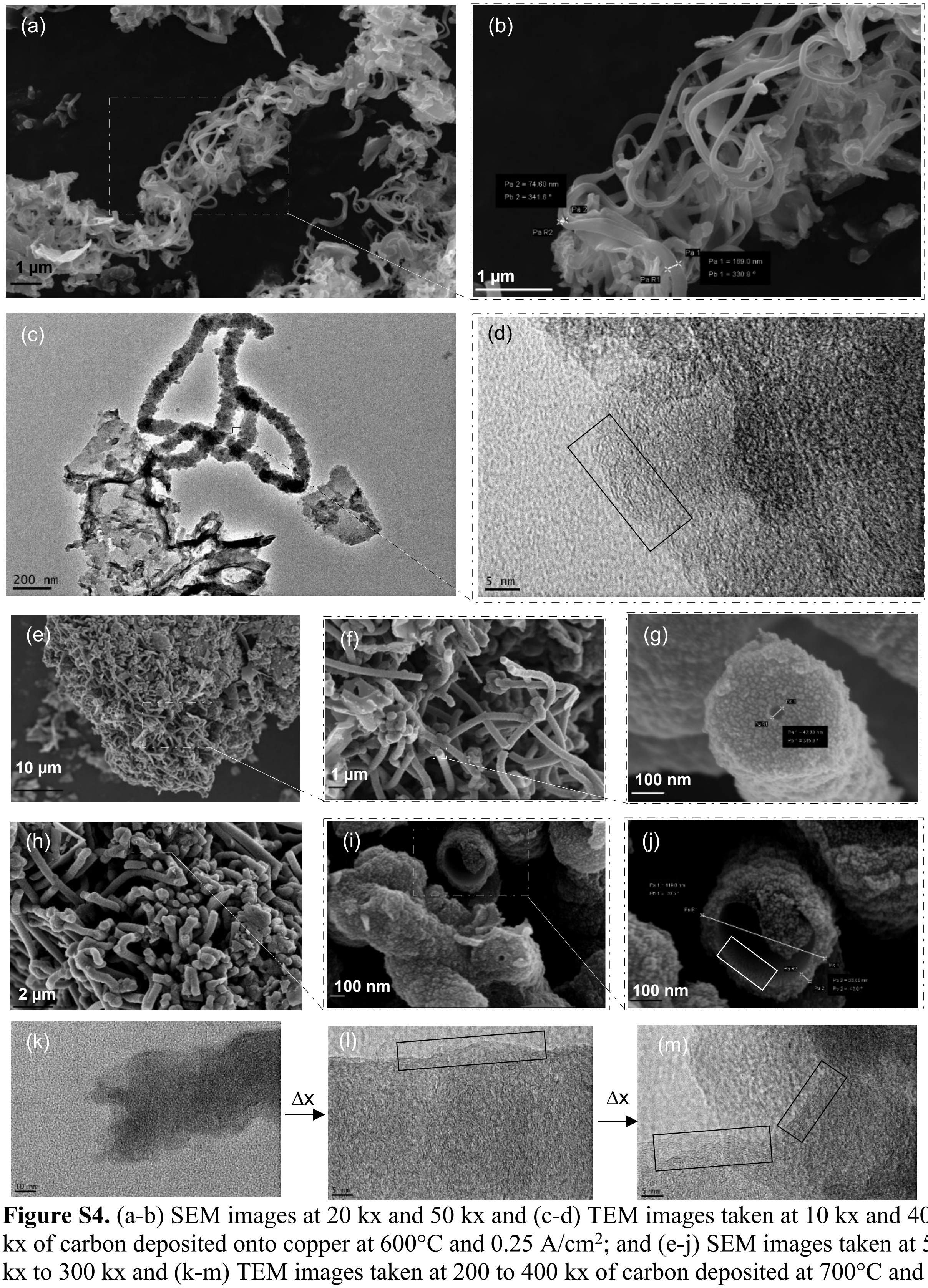

Figure S4. (a-b) SEM images at 20 kx and 50 kx and (c-d) TEM images taken at 10 kx and 400 kx of carbon deposited onto copper at 600°C and 0.25 A/cm^2; and (e-j) SEM images taken at 5 kx to 300 kx and (k-m) TEM images taken at 200 to 400 kx of carbon deposited at 700°C and 0.15 A/cm^2.

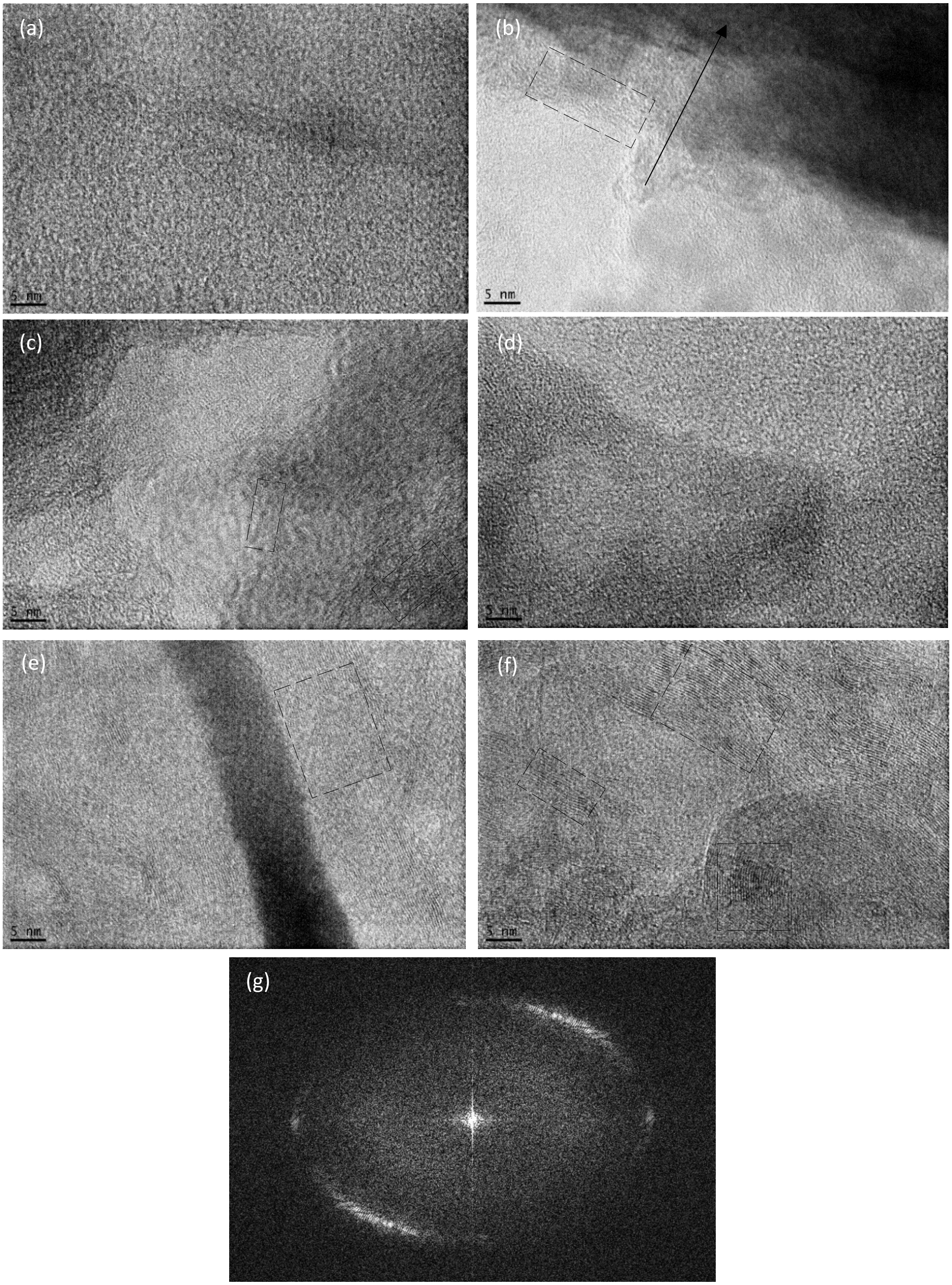

Figure S5. TEM images taken at 400 000 x magnification of carbon deposited at (a-b) 500°C, (c-d) 600°C and (e-f) 700°C, and (g) the diffraction pattern of the carbon sheet shown in (f).

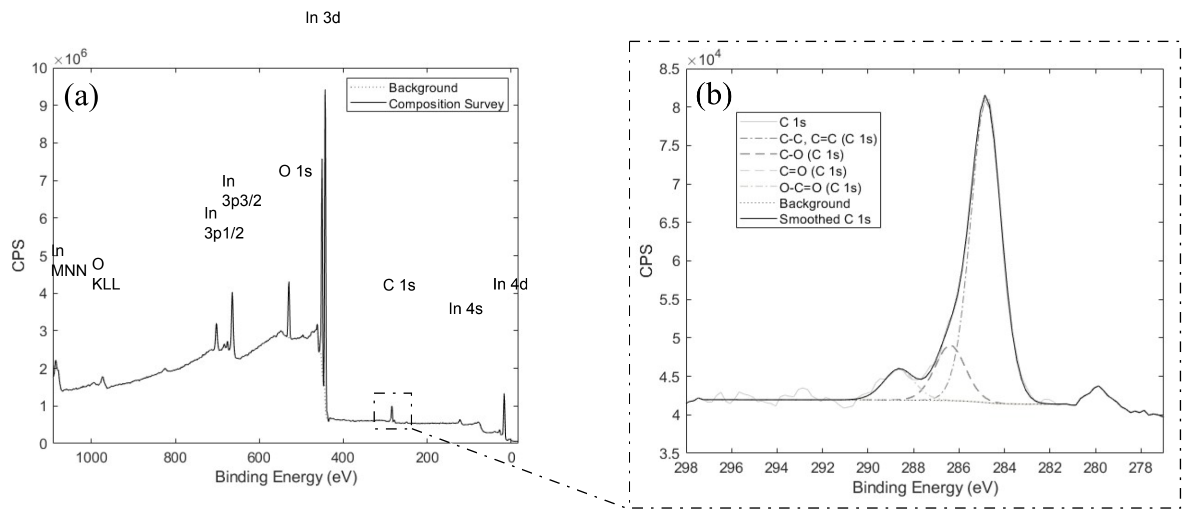

Figure S6. The XPS (a) survey scan and (b) C 1s region scan for indium foil of the type used when mounting carbons derived through the electrolytic reduction of molten carbonate salts for XPS studies.

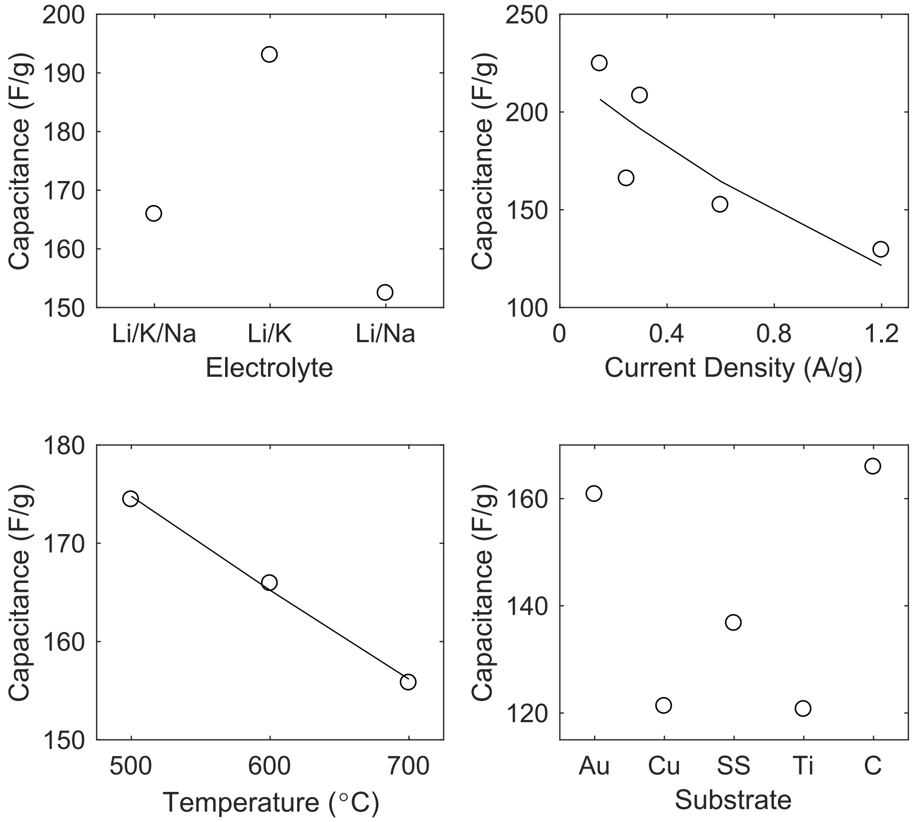

Figure S7. The average capacitances of carbons synthesised through molten carbonate reduction (a) in different molten carbonates, (b) at different current densities, (c) at different temperatures and (d) using different substrates under variations on the standard conditions. Capacitance calculations were based on CV at 25 mV/s in 0.5 M H_2SO_4.

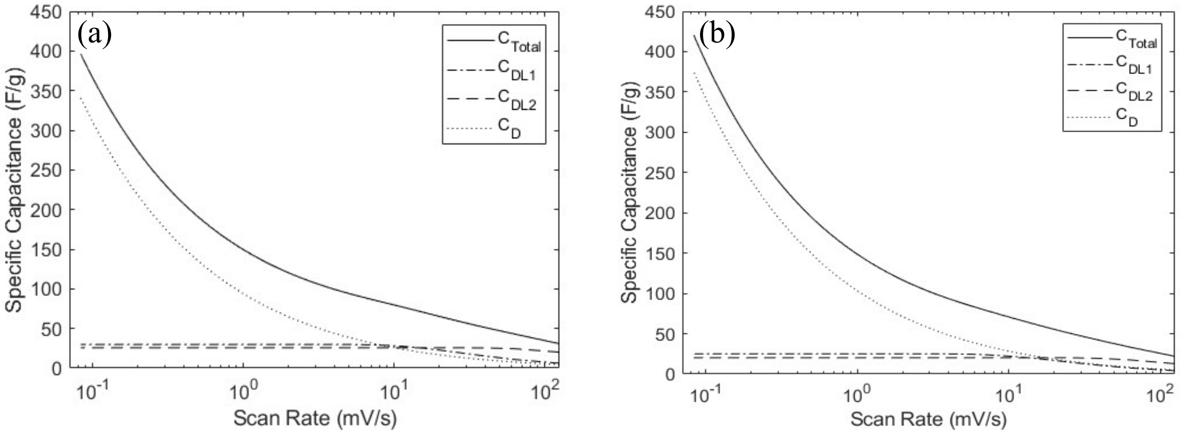

Figure S8. The influence of scan rate on the contributions to the total capacitance of (a) carbon deposited at 1.20 A.cm^{-2} and otherwise standard conditions, and (b) carbon deposited at 700°C under otherwise standard conditions. C_D represents capacitance due to diffusion, while C_{DL1} and C_{DL2} represent the double layers associated with the nano and microporous surface area and the external carbon surface area respectively.
